# Supplementary material for: Inhibition of Endosteal Vascular Niche Remodeling Rescues Hematopoietic Stem Cell Loss in AML
Source: Cell Stem Cell. 2018 Jan 4;22(1):64–77.e6. doi: 10.1016/j.stem.2017.11.006 (PMC5766835; doi:10.1016/j.stem.2017.11.006)
Supplement: Document S1. Figures S1–S7 [file mmc1.pdf]

Cell Stem Cell, Volume 22

Supplemental Information

Inhibition of Endosteal Vascular Niche Remodeling Rescues Hematopoietic Stem Cell Loss in AML

Delfim Duarte, Edwin D. Hawkins, Olufolake Akinduro, Heather Ang, Katia De Filippo, Isabella Y. Kong, Myriam Haltalli, Nicola Ruivo, Lenny Straszkowski, Stephin J. Vervoort, Catriona McLean, Tom S. Weber, Reema Khorshed, Chiara Pirillo, Andrew Wei, Saravana K. Ramasamy, Anjali P. Kusumbe, Ken Duffy, Ralf H. Adams, Louise E. Purton, Leo M. Carlin, and Cristina Lo Celso

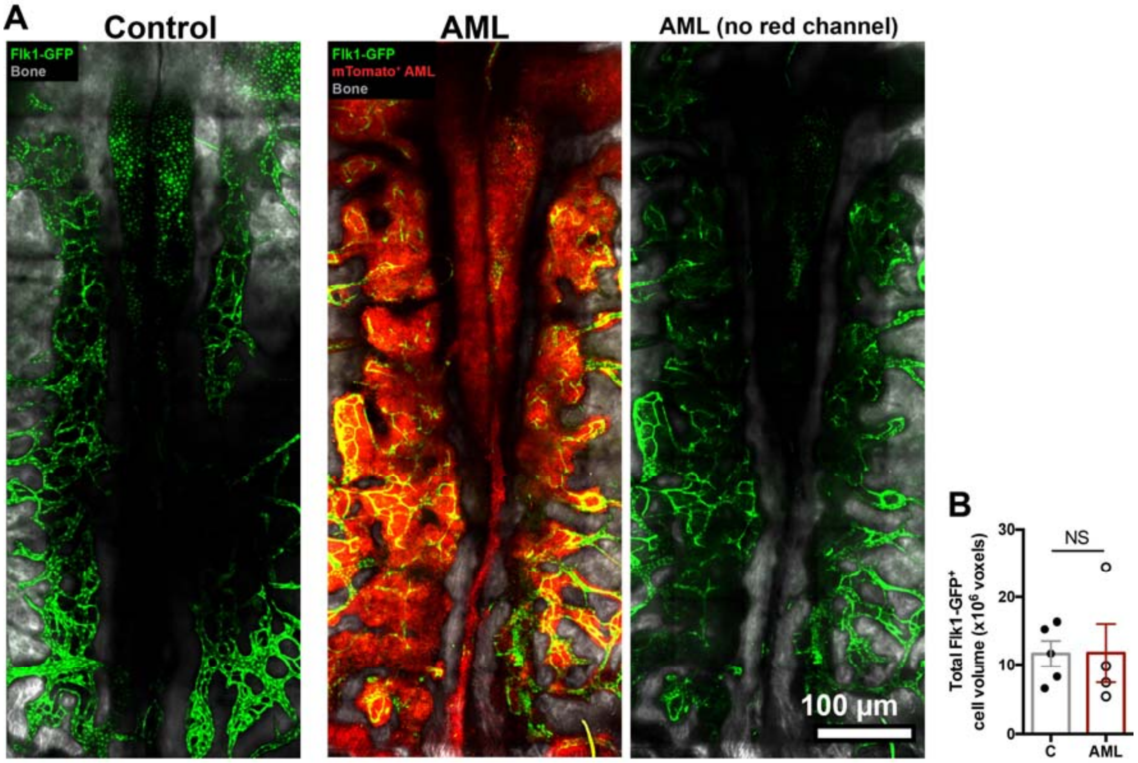

Figure S1 – Intravital imaging of Flk1-GFP mice, related to Figure 2. (A) Representative tilescans (composite of individual tiles) of control and leukemic Flk1-GFP mice. Green: Flk1-GFP^{+} cells (maximum projection); Grey: bone collagen SHG (median projection); Red: mTomato^{+} leukemia cells. **(B)** Total Flk1-GFP^{+} cell volume from acquired tilescans. Data are from 5 control and 4 AML mice.

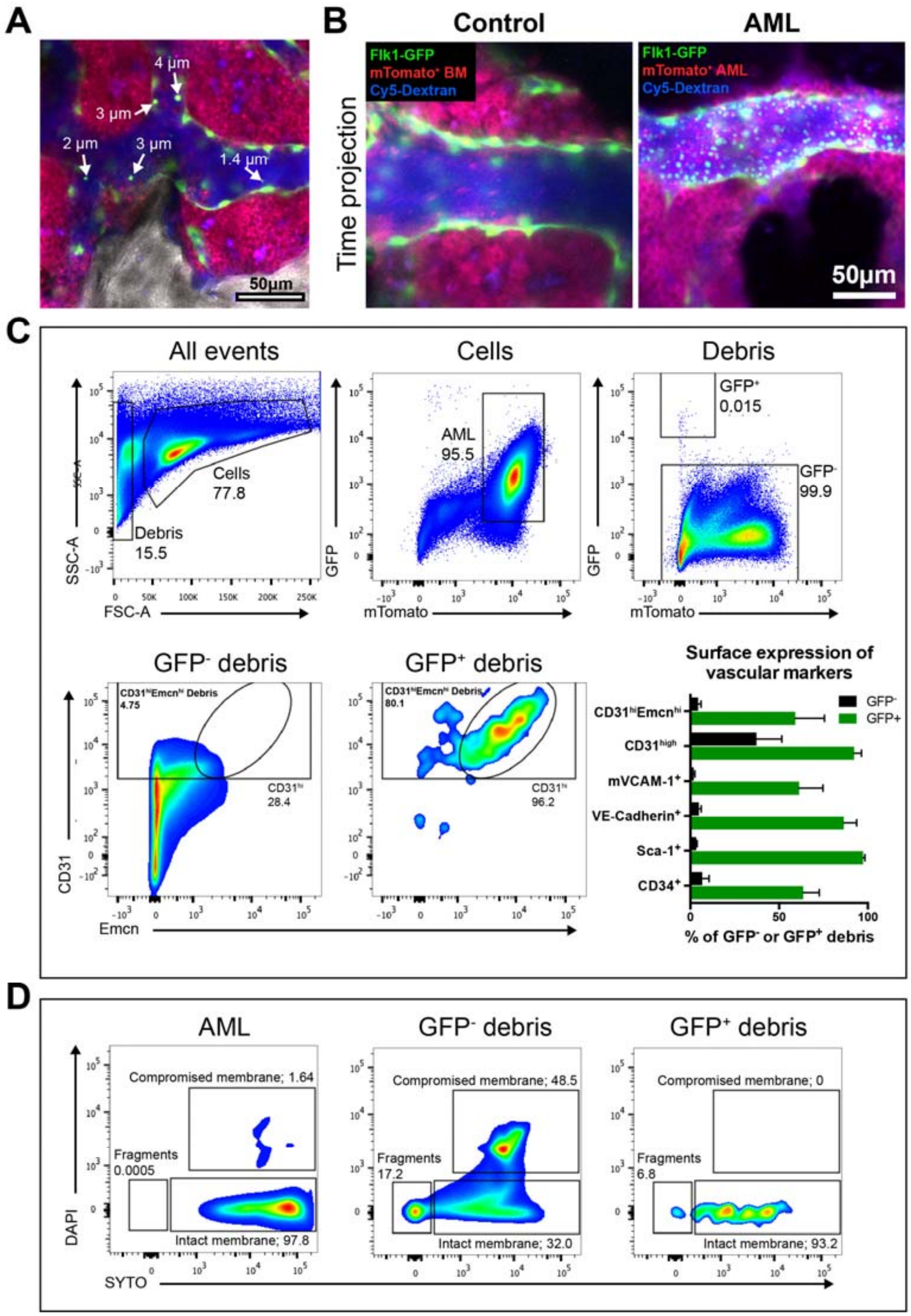

Figure S2 – Cellular debris found in circulation in Flk1-GFP$^+$ AML-burdened mice, related to Figure 2. (A, B) IVM images of GFP$^+$ debris found in circulation in leukemia-burdened, but not healthy Flk1-GFP mice (A: single 2D frame; B: time projection of Movie S3). **(C)** Gating strategy used to identify GFP$^+$ debris, expressing high levels of vascular markers, as assessed by flow cytometry of the BM. **(D)** GFP$^+$ debris is positive for the cell-permeable SYTO and negative for the cell-impermeable DAPI dyes, suggesting that they are membrane-layered particles, carrying variable levels of nucleic acids. Data representative of 3 leukemic mice.

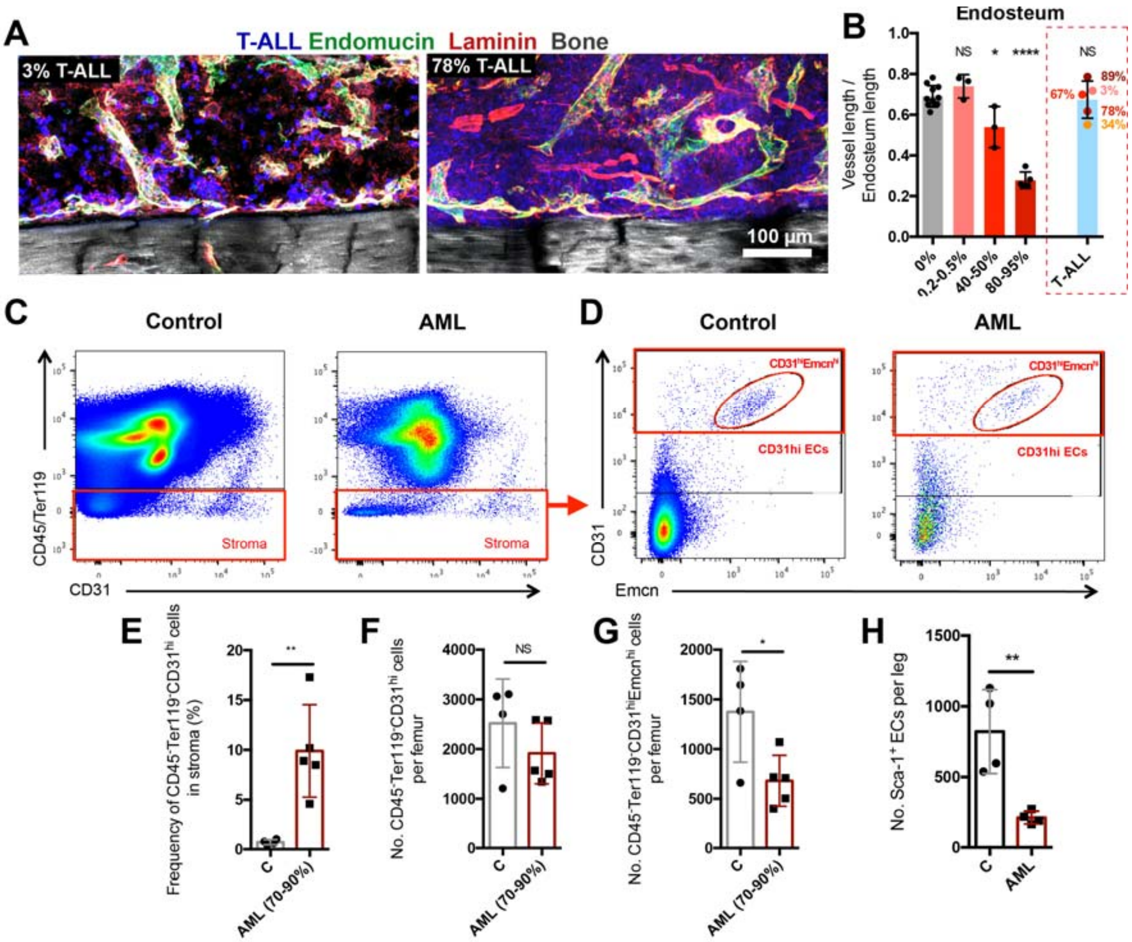

Figure S3 – Endosteal vessels are maintained in T-ALL but decreased in AML-burdened mice, related to Figure 3. (A) Maximum intensity projections of representative immunostained sections comparing endosteal vessels in mice poorly (3%) and highly (78%) infiltrated with T-ALL. Grey: bone collagen SHG; green: endomucin^{+} vessels; red: laminin^{+} vessels and extracellular matrix; blue: T-ALL cells. **(B)** Quantification of endosteal vessels in mice with different levels of T-ALL (each dot represents a mouse). Control and AML samples are reproduced from Figure 3C. **(C, D)** Gating strategy used to quantify BM stroma cells **(C)** and ECs **(D)** by flow cytometry. Although the frequency **(E)** of ECs is increased, absolute EC numbers **(F)** are unaltered in AML-burdened mice. Absolute numbers of **(G)** CD31^{hi}Emcn^{hi} and **(H)** CD31^{+}Sca-1^{+} endosteal ECs are significantly decreased in fully infiltrated mice. Data obtained from 4 control and 5 leukemic mice (C-G) and from 4 control and 4 leukemic mice (H). Error bars: mean ± SEM.

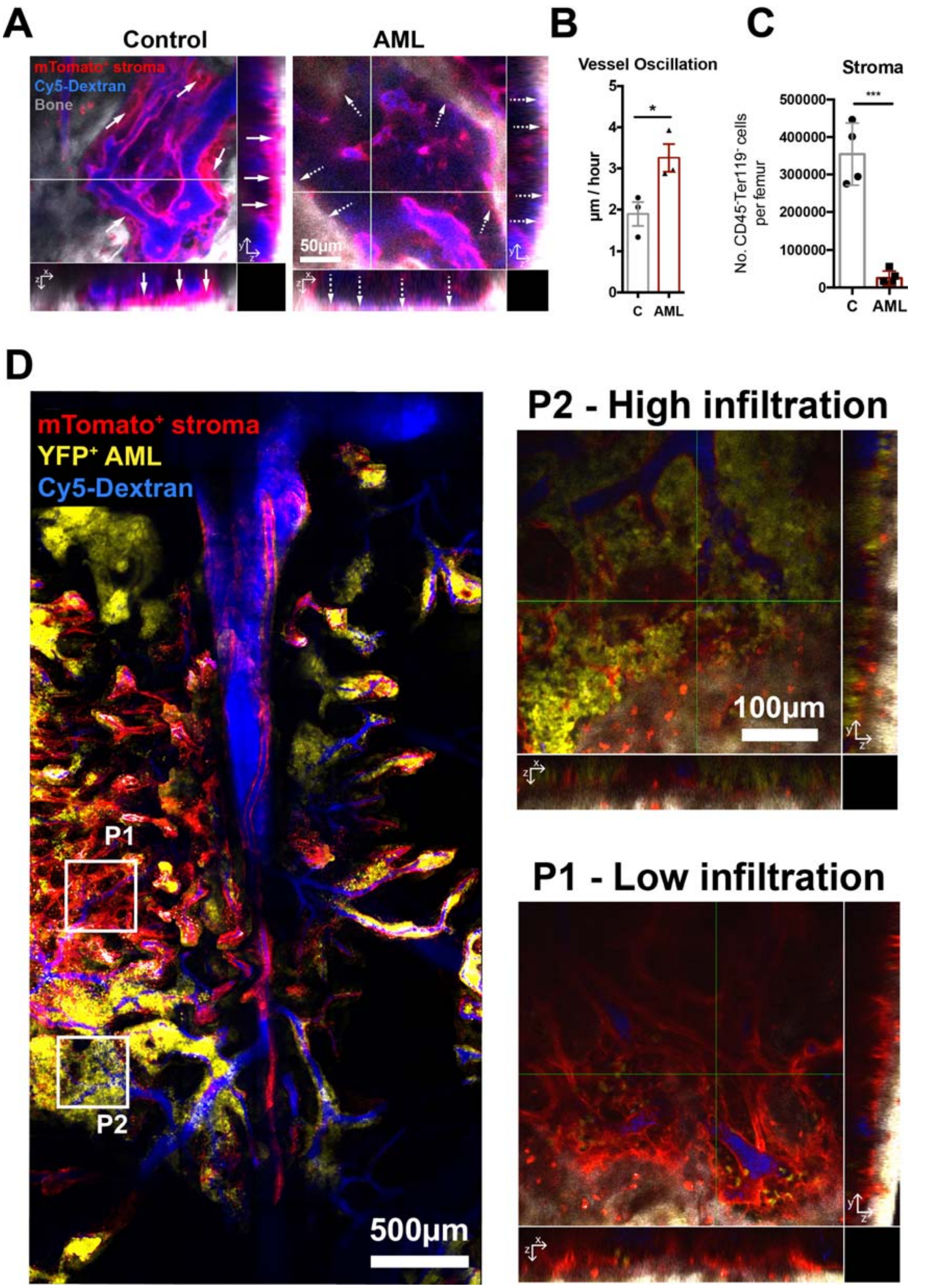

Figure S4 – Stroma is locally and progressively remodeled in AML, related to Figure 3. (A) Maximum intensity projections and orthogonal views of representative BM areas of mT/mG mice reconstituted with non-fluorescent wild-type BM and subsequently transplanted with YFP$^+$GFP$^+$ leukemia cells (AML, cells not shown).

AML-burdened mice show depletion of $mTomato^+$ stromal cells (red) adjacent to vessels (Cy-5 dextran; blue) and bone (SHG; grey). Arrows point at endosteal stroma in control mice. Dashed arrows evidence the loss of endosteal stroma in AML. Data representative of 3 control and 3 leukemic mice. YFP^+ AML cells not shown for clarity purposes. **(B)** Vascular oscillation is significantly increased in mice with AML, as shown in the Movie S4. Data pooled from a total of 2 BM positions per mouse, obtained from 3 control and 3 leukemic mice. Error bars: mean ± SEM **(C)** Absolute numbers of overall $CD45^-Ter119^-$ stromal cells are significantly decreased in AML-burdened mice. Data obtained from non-chimeric 4 control and 5 leukemic mice. *** $p<0.001$. Error bars: mean ± SD. **(D)** Representative tilescan (composite of individual tiles) and selected P1 and P2 areas showing AML cells (yellow) colonizing the calvarium in clusters and locally depleting mTomato+ stromal cells (red) adjacent to blood vessels (Cy5 dextran; blue) and bone (SHG; grey). Data representative of 3 mice.

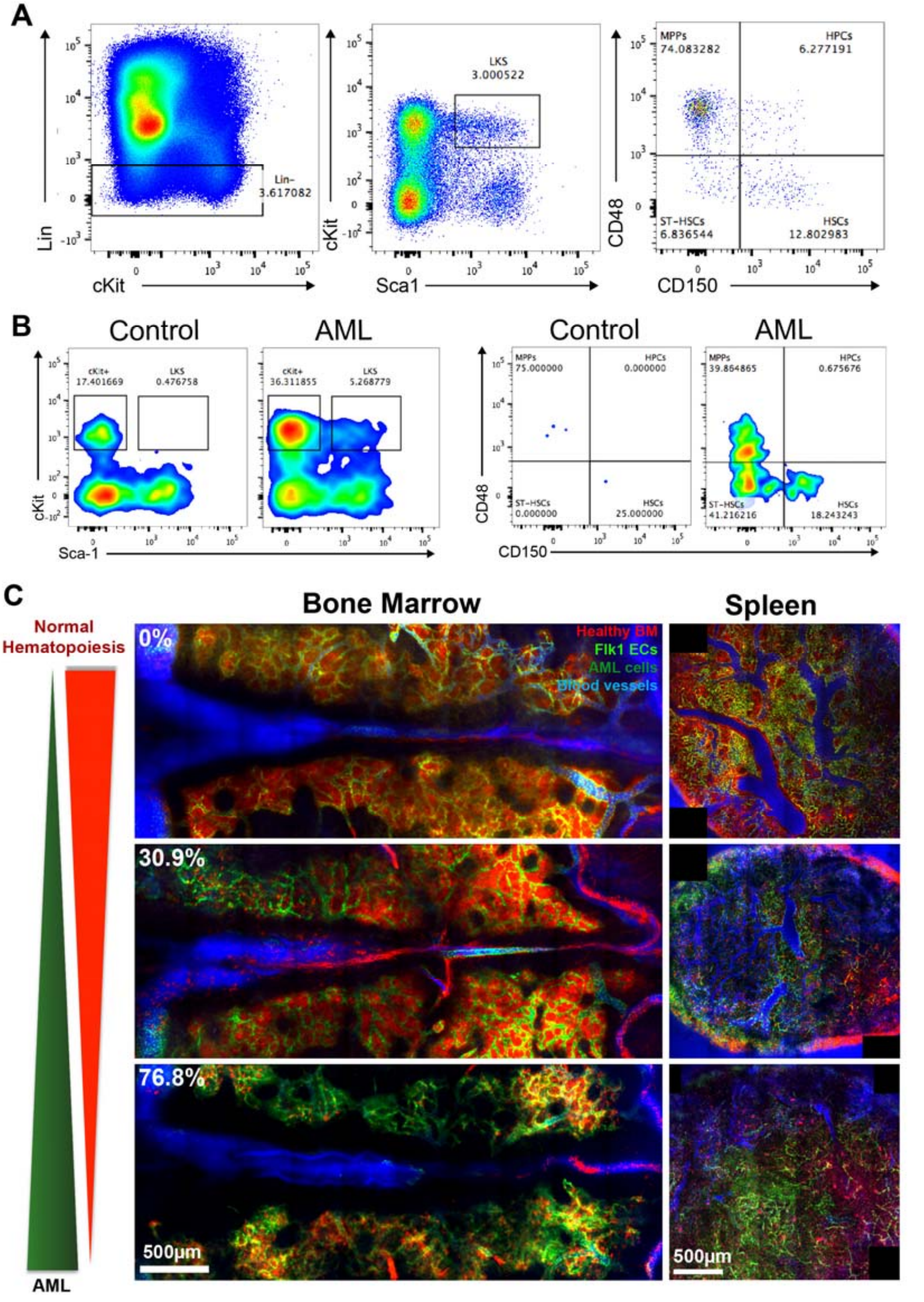

Figure S5 – Hematopoiesis loss in the BM is coupled with extramedullary hematopoiesis in the spleen, related to Figure 5. (A) Gating strategy used to identify healthy Lineage$^-$ cKit$^+$ Sca-1$^+$ (LKS) progenitors and LKS CD48$^-$ CD150$^+$ hematopoietic stem cell (HSCs). **(B)** Flow cytometry analysis reveals emergence of LKS and HSC populations in the leukemic spleen. **(C)** Intravital imaging of BM and spleen. Maximum intensity projections of representative BM and spleen tilescans

(composite of individual tiles) of Flk1-GFP mouse chimeras reconstituted with mTomato$^+$ hematopoietic cells and transplanted with GFP^{low} AML cells. In the case of spleen tilescans, some peripheral tiles are not included in the composite. mTomato$^+$ hematopoietic cells are lost with increasing levels of AML infiltration. For each mouse, BM and spleen were imaged on the same day. To track single mTomato$^+$ healthy hematopoietic cells in both the BM and spleen, we performed time-lapse imaging with high temporal resolution (30s acquisition interval) – see Figure 5. Green: Flk1-GFP$^+$ ECs; red: mTomato$^+$ healthy hematopoietic cells; blue: Cy5 Dextran.

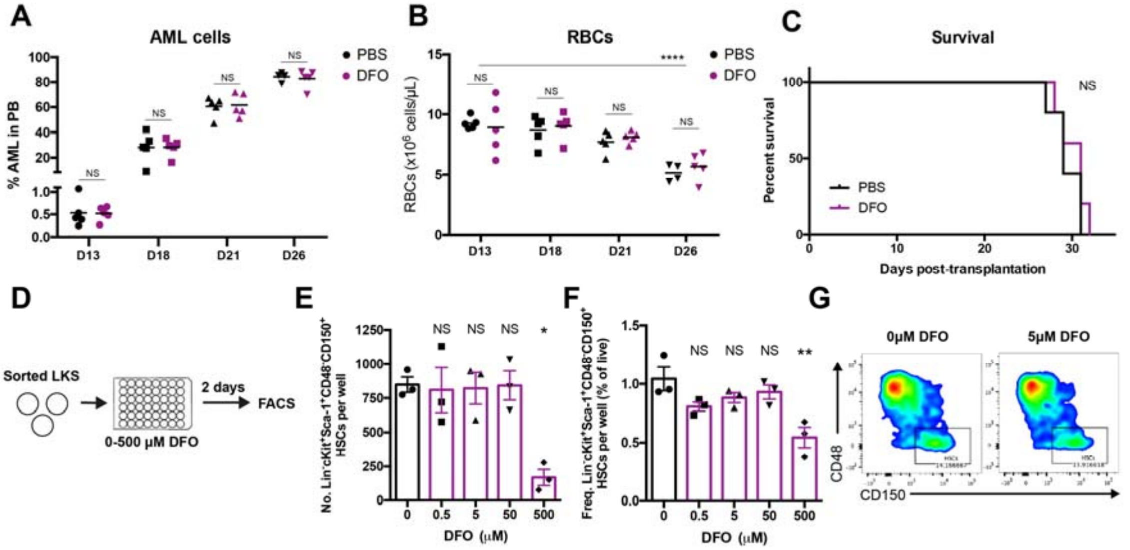

Figure S6 – DFO effect on AML progression *in vivo* and HSCs *in vitro*, related to Figure 6. Mice transplanted with AML were treated with either PBS or DFO from day 8 post-transplantation. **(A)** Percentage of mTomato$^+$ blasts and **(B)** red blood cell (RBC) counts in the peripheral blood and **(C)** survival of DFO- and PBS-treated mice show similar disease progression in the two groups. Data obtained from 5 control (PBS) and 5 DFO-treated mice. **(D)** Live Lin$^-$cKit$^+$Sca-1$^+$ (LKS) cells were sorted, plated and incubated with increasing concentrations of DFO. 2 days later the **(E)** number and **(F, G)** frequency of Lin$^-$cKit$^+$Sca-1$^+$CD48$^-$CD150$^+$ HSCs were assessed by FACS. Data shown are representative of three independent experiments.

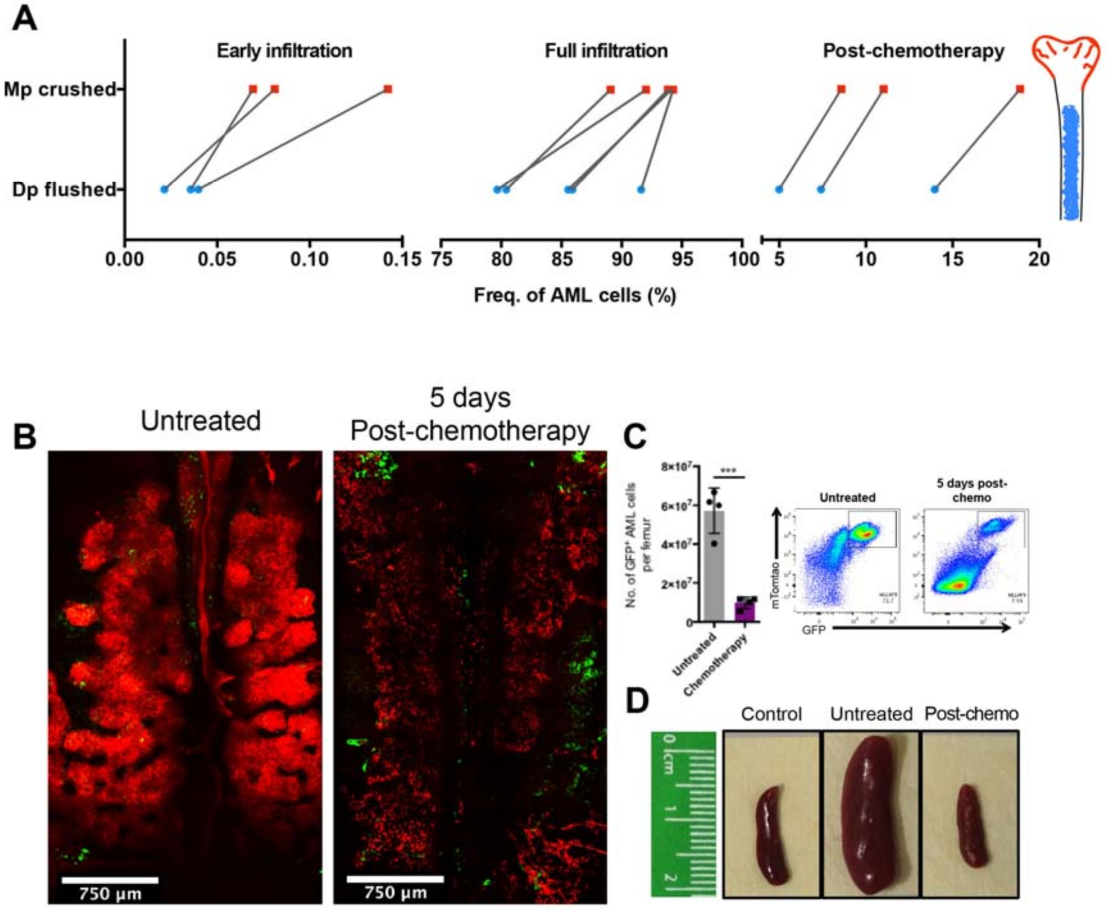

Figure S7 – Experimental model of AML induction chemotherapy, related to Figure 7. (A) Paired comparison shows that leukemic cells are more frequent in crushed metaphysis (Mp) than in flushed diaphysis (Dp) at very early (3 mice) and late (5 mice) infiltration and post-chemotherapy (3 mice). **(B)** Maximum projection tilescan (composite of individual tiles) of calvarium BM of Col2.3-GFP mice (green: osteoblastic cells) carrying mTomato$^+$ AML cells (red) before and after chemotherapy (3 days of cytarabine and doxorubicin followed by 2 days of cytarabine alone). **(C)** Flow cytometry quantification of absolute numbers of AML cells in the BM. Data obtained from 4 control and 4 leukemic mice. Error bars: mean ± S.D. **(D)** Appearance of spleens from control, untreated and 5 days-treated mice.

Supplementary movies legends

Movie S1 – Inefficient angiogenesis in AML-burdened mice. Representative maximum projection of 3D time-lapse data (shown at 10 frames per second) of an area from a control (left) and leukemic (right) Flk1-GFP mouse collected every 90s for 90min (shown in figure 2G). Red arrowheads point to vascular sprouts that rapidly retract. Black: Flk1-GFP$^+$ cells. Representative of 4 control and 3 leukemic mice.

Movie S2 – Blood vessel fragmentation in mice with AML. Maximum projection of 3D time-lapse data (shown at 10 frames per second) of vascular disintegration (circled area; red arrows) in a leukemic Flk1-GFP mouse. Filmed every 90s for 90min. Black: Flk1-GFP$^+$ cells. Equivalent events were never observed in control mice.

Movie S3 – Circulating endothelial debris in BM vessels of AML-burdened mice. Representative 2D time-lapse data collected every 156ms for 4min 24s (shown at 5 frames per second) from a Flk1-GFP mouse reconstituted with mTomato$^+$ healthy hematopoietic cells (left; Control) and a Flk1-GFP mouse infiltrated with mTomato$^+$ AML (right; AML). In control mice (left) no debris particles are detected in circulation but in leukemic mice (right) frequent endothelial debris is found inside the vascular lumen, sometimes adhering to the endothelium. Green: GFP signal; red: mTomato$^+$ healthy hematopoietic cells (left) or AML cells (right); blue: Cy5-Dextran. Arrowheads follow some of the debris observed in circulation. Representative of 3 control and 4 leukemic mice.

Movie S4 - Stroma dynamics in mice with AML. Representative maximum projections of 3D time-lapse data (shown at 10 frames per second) collected at ten-minute intervals for 7h and 20min from a mT/mG control (left) chimera and a mT/mG chimera with high infiltration of GFP$^+$YFP$^+$ AML (right). AML cells not shown for clarity purposes. Red: mTomato$^+$ stromal cells; blue: Cy-5 dextran$^+$ blood vessels. Arrows follow oscillating vessels in AML-burdened mouse. Representative of 3 control and 3 leukemic mice.

Movie S5 – Cell adhesion to the splenic endothelium. Representative maximum projections of time-lapse data (shown at 7 frames per second) of 2 areas scanned every 30s for 15min from the spleen of a leukemic Flk1-GFP mouse with mTomato$^+$ residual healthy hematopoietic cells. In area 1, the arrow points to a healthy hematopoietic cell adhering statically to the endothelium. In position 2, a cell adheres, crawls and detaches from the endothelium. Green: Flk1$^+$ GFP ECs; red: mTomato$^+$ healthy hematopoietic cells; blue: Cy5-Dextran.

Movie S6 – Transendothelial migration in the bone marrow. Representative maximum projections of time-lapse data (shown at 7 frames per second) of 2 areas scanned every 30s for 15min from the BM of a leukemic Flk1-GFP mouse with mTomato$^+$ residual healthy hematopoietic cells. In position 1, the arrow points to a normal hematopoietic cell intravasating and leaving the BM. In position 2, a cell adheres and extravasates towards the tissue. Green: Flk1$^+$ GFP ECs; red: mTomato$^+$ non-malignant hematopoietic cells; blue: Cy5-Dextran.

Movie S7 – Turbulent blood flow in vessels within AML infiltrated BM. Representative maximum projection (shown at 7 frames per second) of a vascular

bifurcation area collected every 30s for 15min from the spleen of a healthy (left) and a leukemic (right) Flk1-GFP mouse. Dark circles: AML cells form intravascular clusters that adhere to the endothelium and block blood flow; Green: $Flk1^+$ GFP ECs; Red: $mTomato^+$ non-malignant hematopoietic cells; Yellow: Cy5-Dextran.
