# Supplementary material for: Inhibition of Endosteal Vascular Niche Remodeling Rescues Hematopoietic Stem Cell Loss in AML
Source: Cell Stem Cell. 2018 Jan 4;22(1):64–77.e6. doi: 10.1016/j.stem.2017.11.006 (PMC5766835; doi:10.1016/j.stem.2017.11.006)
Supplement: Document S2. Article plus Supplemental Information [file mmc9.pdf]

Article

Cell Stem Cell

Inhibition of Endosteal Vascular Niche Remodeling Rescues Hematopoietic Stem Cell Loss in AML

Graphical Abstract

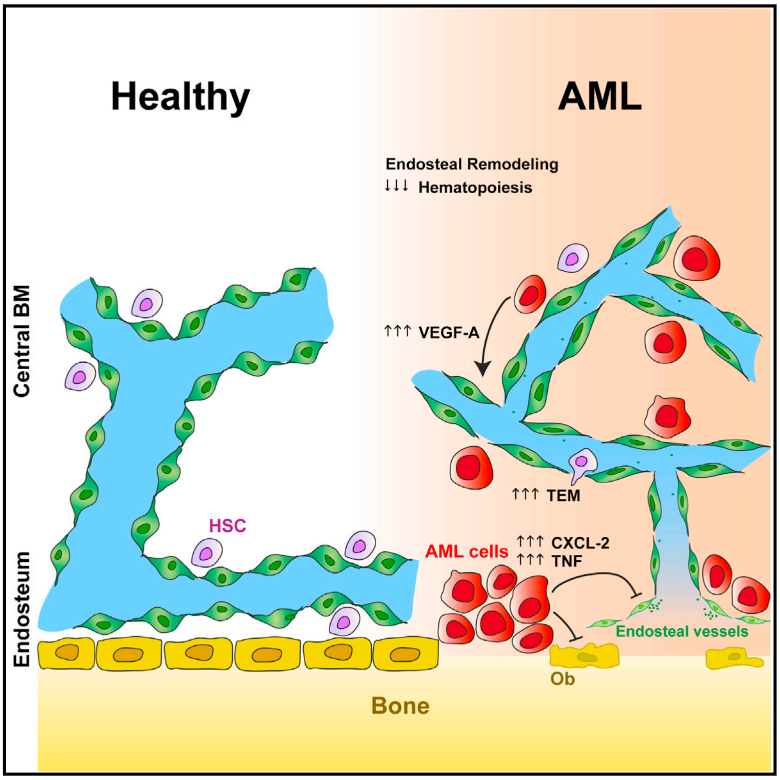

Highlights

- AML leads to progressive remodeling of endosteal stroma
- HSC loss is spatiotemporally correlated with endosteal remodeling
- *In vivo* imaging reveals transendothelial migration of healthy hematopoietic cells
- Rescue of endosteal vessels preserves HSCs and enhances the efficacy of chemotherapy

Authors

Delfim Duarte, Edwin D. Hawkins, Olufolake Akinduro, ..., Louise E. Purton, Leo M. Carlin, Cristina Lo Celso

Correspondence

delfimd@med.up.pt (D.D.), c.lo-celso@imperial.ac.uk (C.L.C.)

In Brief

Multi-modal microscopy of acute myeloid leukemia progression within the bone marrow reveals focal and progressive remodeling of endosteal blood vessels coupled to loss of osteoblasts, hematopoietic stem cells (HSCs), and HSC niches. Preserving endosteal vessels increases the number of surviving HSCs and improves the efficacy of chemotherapy.

Duarte et al., 2018, Cell Stem Cell *22*, 64–77
January 4, 2018 © 2017 The Authors. Published by Elsevier Inc.
https://doi.org/10.1016/j.stem.2017.11.006

CellPress

Cell Stem Cell
Article

Inhibition of Endosteal Vascular Niche Remodeling Rescues Hematopoietic Stem Cell Loss in AML

Delfim Duarte,[1,2,*] Edwin D. Hawkins,[1,3,4] Olufolake Akinduro,[1] Heather Ang,[1] Katia De Filippo,[5] Isabella Y. Kong,[3,4] Myriam Haltalli,[1] Nicola Ruivo,[1] Lenny Straszkowski,[6] Stephin J. Vervoort,[7,8] Catriona McLean,[9] Tom S. Weber,[10,17] Reema Khorshed,[1] Chiara Pirillo,[1] Andrew Wei,[9] Saravana K. Ramasamy,[11] Anjali P. Kusumbe,[12] Ken Duffy,[10] Ralf H. Adams,[13,14] Louise E. Purton,[6,15] Leo M. Carlin,[5,16] and Cristina Lo Celso[1,2,18,*]
[1]Department of Life Sciences, Sir Alexander Fleming Building, Imperial College London, SW7 2AZ London, UK
[2]The Francis Crick Institute, WC2A 3LY London, UK
[3]The Walter and Eliza Hall Institute of Medical Research, Melbourne, VIC 3052, Australia
[4]Department of Medical Biology, The University of Melbourne, Parkville, VIC 3010, Australia
[5]Inflammation, Repair and Development, National Heart and Lung Institute, Imperial College London, SW7 2AZ London, UK
[6]Stem Cell Regulation Unit, St. Vincent's Institute of Medical Research, Fitzroy, VIC 3065, Australia
[7]Sir Peter MacCallum Department of Oncology, University of Melbourne, Parkville, VIC 3052, Australia
[8]Peter MacCallum Cancer Centre, Melbourne, VIC 3000, Australia
[9]Department of Haematology, Alfred Hospital, Melbourne, VIC 3004, Australia
[10]Hamilton Institute, Maynooth University, Maynooth, Ireland
[11]Institute of Clinical Sciences, Imperial College London, W12 0NN London, UK
[12]Kennedy Institute of Rheumatology, Nuffield Department of Orthopaedics, Rheumatology and Musculoskeletal Sciences, University of Oxford, Headington, Oxford OX3 7FY, UK
[13]Max Planck Institute for Molecular Biomedicine, Department of Tissue Morphogenesis, 48149 Munster, Germany
[14]University of Münster, Faculty of Medicine, 48149 Munster, Germany
[15]Department of Medicine, The University of Melbourne, Fitzroy, VIC 3065, Australia
[16]Cancer Research UK Beatson Institute, Glasgow G61 1BD, UK
[17]Present address: The Walter and Eliza Hall Institute of Medical Research, Melbourne, VIC 3052, Australia
[18]Lead Contact
*Correspondence: delfimd@med.up.pt (D.D.), c.lo-celso@imperial.ac.uk (C.L.C.)
https://doi.org/10.1016/j.stem.2017.11.006

SUMMARY

Bone marrow vascular niches sustain hematopoietic stem cells (HSCs) and are drastically remodeled in leukemia to support pathological functions. Acute myeloid leukemia (AML) cells produce angiogenic factors, which likely contribute to this remodeling, but anti-angiogenic therapies do not improve AML patient outcomes. Using intravital microscopy, we found that AML progression leads to differential remodeling of vasculature in central and endosteal bone marrow regions. Endosteal AML cells produce pro-inflammatory and anti-angiogenic cytokines and gradually degrade endosteal endothelium, stromal cells, and osteoblastic cells, whereas central marrow remains vascularized and splenic vascular niches expand. Remodeled endosteal regions have reduced capacity to support non-leukemic HSCs, correlating with loss of normal hematopoiesis. Preserving endosteal endothelium with the small molecule deferoxamine or a genetic approach rescues HSCs loss, promotes chemotherapeutic efficacy, and enhances survival. These findings suggest that preventing degradation of the endosteal vasculature may improve current paradigms for treating AML.

INTRODUCTION

Hematopoietic stem cells (HSCs) reside in the bone marrow (BM), where they receive survival and differentiation signals from several cell types, including endothelial and multiple lineages of peri-vascular mesenchymal cells (Morrison and Scadden, 2014). Similarly, cancer growth and chemo-resistance have been hypothesized to be dependent on a malignant micro-environment that is highly vascularized (Duan et al., 2014; Pitt et al., 2015). This relationship is well illustrated in epithelial cancers, where increased angiogenesis supports growth and metastasis (Quail and Joyce, 2013).

Acute myeloid leukemia (AML) is an aggressive leukemia often accompanied by life-threatening cytopenias. The cure rate of AML is only 5%–15% in patients greater than 60 years old (Döhner et al., 2015). Thus, there is an unmet clinical need for more effective therapies, especially because the mainstay of treatment has not changed significantly in the last 30 years (Döhner et al., 2015). To develop more selective and better-tolerated therapies, it is critical that we understand how AML cells grow, outcompete healthy hematopoiesis, and eventually generate an environment supportive of chemo-resistant leukemia stem cells. Alterations in BM innervation and stroma have been described in late stages of disease (Hanoun et al., 2014). However, the dynamic process leading to this stage is unknown, and its dissection promises to uncover new therapeutic targets. There are reports of vascular endothelial growth factor (VEGF) secretion by AML cells (Fiedler et al., 1997) and of increased

 © 2017 The Authors. Published by Elsevier Inc.
This is an open access article under the CC BY license (http://creativecommons.org/licenses/by/4.0/).

BM microvasculature density in patients (Aguayo et al., 2000; Hussong et al., 2000; Padró et al., 2000) and, more recently, murine models (Hanoun et al., 2014) at advanced stages of disease. However, clinical trials investigating anti-angiogenic therapy in AML patients have been disappointing (Fiedler et al., 2003; Ossenkoppele et al., 2012; Zahiragic et al., 2007). Thus, questions remain about the effect of AML growth on BM vasculature and whether vascular remodeling may be beneficial for the disease. We hypothesized that AML-induced BM vascular remodeling may be more complex than simple induction of angiogenesis and that progressive, nuanced changes could shape the ecological competition between leukemia and healthy hematopoiesis. A spatiotemporal understanding of these changes may point to novel candidate interventions that could restore BM normal ecology, including HSC function, and in turn make AML cells more susceptible to chemotherapy.

Here, we present a high-resolution, longitudinal analysis of the progressive and region-specific vasculature remodeling induced by AML, with a particular focus on the implications for healthy hematopoiesis. We uncovered (1) morphological and functional changes in surviving vessels, namely attempted but failed angiogenesis and increased transendothelial migration of hematopoietic cells; (2) specific loss of endosteal vessels, accompanied by loss of osteoblastic cells and, most importantly, HSCs; and (3) endosteal vasculature induction as a successful approach to rescue healthy HSCs and improve the efficacy of induction chemotherapy.

RESULTS

Intravital Microscopy Enables the Study of AML Cells, Healthy Hematopoietic Cells, and the BM Microenvironment Simultaneously

To study the effects of AML growth on BM vasculature and HSCs as disease propagates through the tissue, we used the well-established MLL-AF9-driven murine model of AML, which recapitulates phenotypic and pathological features of human MLL-rearranged AML (Krivtsov et al., 2006; Somervaille and Cleary, 2006). To generate leukemia cells detectable by intravital microscopy, we harvested myeloid progenitor cells from donor mice that expressed high levels of fluorescent proteins (FP), transduced them with a retroviral vector encoding the oncogene and GFP, and injected them into sub-lethally irradiated recipients. With this approach, we generated multiple batches of GFP^{+} FP^{+} primary blasts (Figure 1A) that were all $CD11b^{+}$ and contained varying proportions of cells expressing progenitor markers, such as c-Kit, CD34, and FCγRII/III (data not shown). Experiments were repeated using blasts from different primary recipients to ensure identification of consistent features of AML growth and to discount any possible primary donor-specific phenotype. To achieve reliable disease progression while minimizing the number of blasts injected, 100,000 primary AML blasts were transplanted by tail vein injection into non-irradiated secondary recipients. This strategy was critical as potential myelo-ablative conditioning regimens that could favor AML progression and cause hematopoietic and stromal damage were avoided. In all secondary recipients, progressive blast expansion was observed from days 8 to 10 post-transplantation with full BM infiltration typically reached between day 20 and 28, with the variation depending on the source of the primary blasts analyzed. This leukemic engraftment was accompanied by infiltration of the spleen, typically delayed compared to BM infiltration (Figure 1A). In each mouse, healthy hematopoiesis was progressively lost with AML expansion (Figure 1B). AML cells, vasculature, and hematopoietic cells were visualized by intravital microscopy (IVM) performed on mouse calvarium BM (Figure 1C), which has been shown to be representative of long bones' marrow in terms of stroma composition and ability to support functional HSCs and their homing and engraftment (Lassailly et al., 2013; Lo Celso et al., 2009). This approach is minimally invasive and allows longitudinal observation of cellular dynamics (including cell migration, proliferation, and death) taking place within the tissue over the course of hours or days. This approach has been essential for us to uncover previously unappreciated biological processes, such as the ability of HSCs exposed to acute infection to engage wider than normal BM niches (Rashidi et al., 2014), the migratory behavior of T cell acute lymphoblastic leukemia (T-ALL) cells during disease progression and response to chemotherapy, and T-ALL-induced osteoblastic cells remodeling (Hawkins et al., 2016). In particular, tile scan images of the entire BM space contained within the calvarium provide a comprehensive, three-dimensional, single-cell resolution overview of the overall organization of the tissue and are therefore ideal to uncover complex remodeling processes that are still poorly understood.

BM Blood Vessels Are Damaged in AML

To identify progressive changes of blood vessels *in situ* during AML progression, we performed IVM of Flk1-GFP transgenic mice, in which phenotypic endothelial cells (ECs) express GFP (Figure 2A) and can be visualized lining BM blood vessels labeled with Cy5-dextran (Figure 2B). We observed multiple, significant changes in Flk-1 GFP^{+} blood vessels in mice burdened with AML (Figure S1). First, most vessels were narrower than those in control mice (Figure 2C). Second, they were characteristically further from the endosteal bone surface (Figures 2D and 2E). Imaging of partially infiltrated mice was consistent with the findings of (Hérault et al., 2017) in revealing that AML cells clustered in patches of highly infiltrated areas, whereas the remaining BM space contained only sparse AML cells (Figure 2F). Blood vessels in highly infiltrated areas appeared unusually barbed and presented dynamic subcellular protrusions toward the parenchyma (Figure 2F, right panels). High-resolution time-lapse recording of blood vessels at late stages of AML revealed sequential formation and retraction of sprouts (Figure 2G; Movie S1), similar to those described in response to strong angiogenic stimuli (Gerhardt et al., 2003; Jakobsson et al., 2010). However, this sprouting process was never efficient, and we could not detect formation of any steady lateral branches. This is consistent with the increased levels of VEGF-A detected in mice infiltrated with AML (Figure 2H). We also occasionally observed vascular damage caused by EC breakage into small fragments (Movie S2). Consistent with this observation, we observed abundant 1- to 4-μm-sized cellular debris of endothelial origin (GFP^{+}) in the vascular lumen of AML-burdened mice (Figures S2A and S2B; Movie S3). These debris particles maintained expression of phenotypic endothelial markers, including high levels of CD31 and

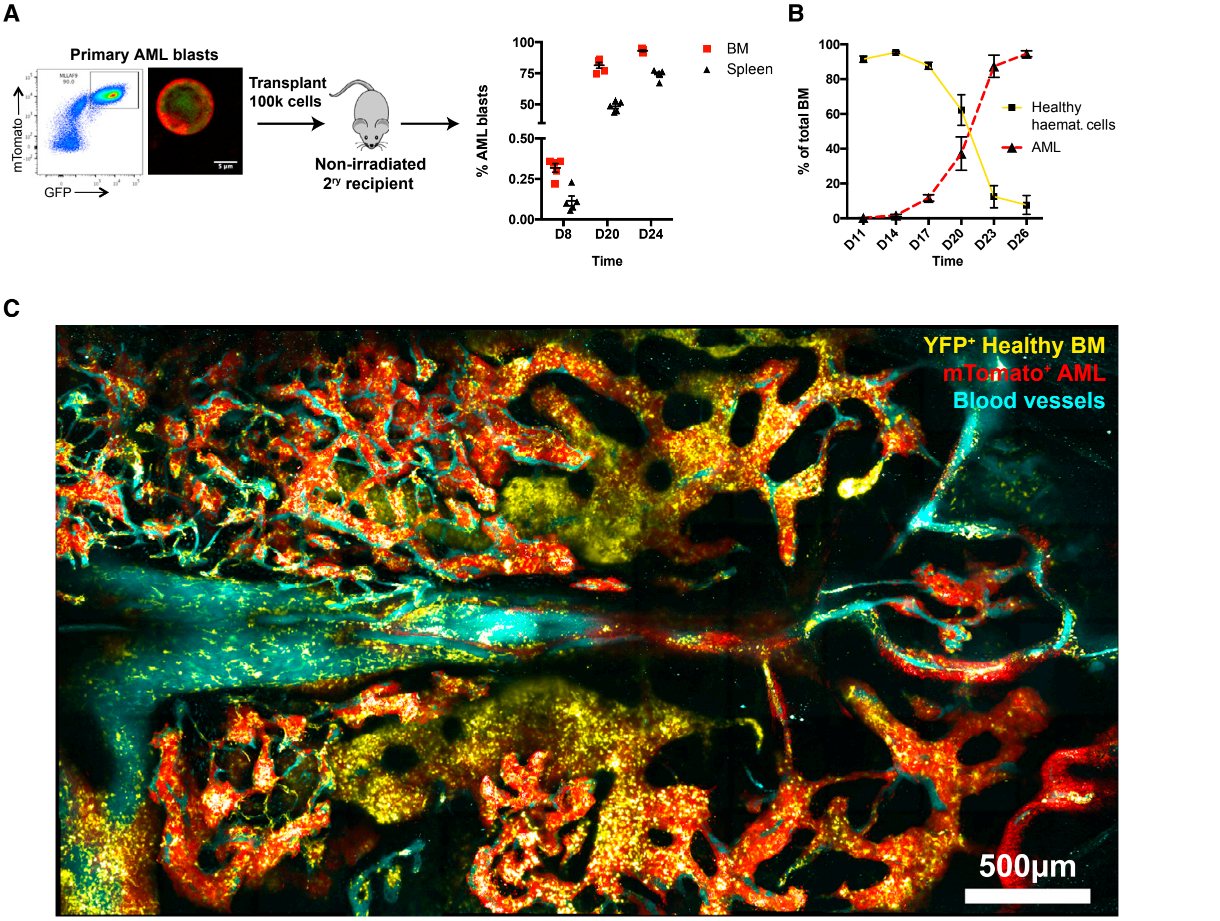

Figure 1. MLL-AF9-Driven Experimental AML Model and Intravital Microscopy

(A) 100,000 primary mTomato (or YFP or non-labeled) and GFP double-positive AML cells are transplanted into non-irradiated secondary recipients, where they progressively infiltrate BM and spleen (n = 5 mice analyzed per time point).

(B) AML blasts infiltrate and outcompete non-malignant, healthy BM cells over time. Data shown are from 5 leukemic mice per time point from 2 independent cohorts. Error bars represent mean ± SD.

(C) Maximum projection of IVM tile scan image (composite of individual tiles) showing AML cells (mTomato$^+$; red) interacting with non-malignant hematopoietic cells (YFP$^{+;}$ yellow) and the vascular microenvironment (Cy5-dextran$^+$ blood vessels; cyan).

endomucin (Figure S2C), and contained nucleic acids within an intact membrane (Figure S2D).

Endosteal Vessels Are Specifically Lost in Mice with AML

Prompted by our initial observations, we performed in-depth analysis of blood vessels in the endosteal areas of long bones using immunofluorescence of whole, undecalcified long bone sections from healthy and diseased mice (Figure 3A). This approach allowed us to simultaneously investigate AML-mediated changes in the vasculature of different BM areas: the central marrow diaphysis; the bone-lining endosteum; and the trabecular metaphysis. We were able to detect a significant decrease of vessels in the endosteum and metaphysis over time (Figures 3B and 3C). The endosteal vessels were significantly, and progressively, lost at both intermediate (40%–50% BM infiltration) and advanced (>80% BM infiltration) disease stages (Figure 3C). Notably, vessel loss was specific to these areas and not observed in the diaphysis region, where vessels were either maintained or transiently increased (Figures 3B and 3C). To investigate the relevance of these observations in humans, we performed additional histological analysis of BM trephine biopsies from AML patients with >80% infiltration and confirmed that endosteal vessels were decreased (Figures 3D and 3E). Additionally, endosteal vessels were maintained in a murine model of Notch-driven T-ALL (Figures S3A and S3B), suggesting that the vascular remodeling we observed is specific to AML. These findings pointed to a specific depletion of the functionally unique endosteal endothelium, recently shown to regulate osteogenesis (Kusumbe et al., 2014) and to maintain HSCs (Itkin et al., 2016; Kusumbe et al., 2016).

Because we observed differential remodeling of the microenvironment in AML-burdened mice, we questioned whether they could be triggered by regional variations in leukemia cells. To address this question, we performed RNA sequencing (RNA-seq) analysis on sorted AML cells from trabecular-rich areas (crushed metaphysis) or central BM areas (flushed diaphysis; Figure 3F). We compared the transcriptome of endosteal and

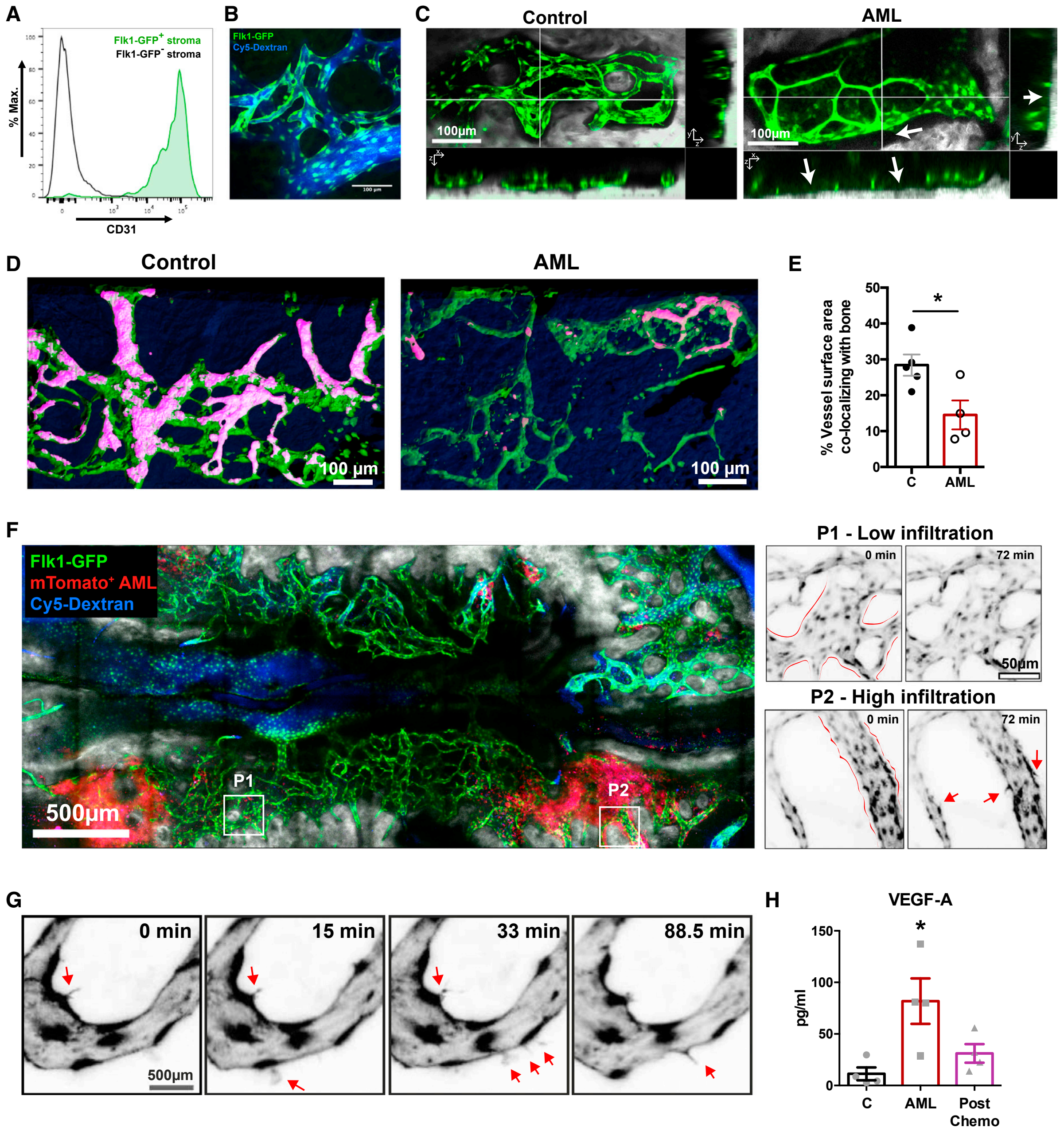

Figure 2. Intravital Imaging of the BM Reveals Blood Vessel Remodeling in AML

(A) Flk1-GFP+ stromal cells (green) express high levels of CD31.

(B) Representative maximum projection of a calvarial area showing Flk1-GFP+ ECs lining blood vessels highlighted by Cy-5-dextran. Blue, Cy-5-dextran; Green, Flk1-GFP+ cells.

(C) Representative maximum projections and respective orthogonal views of Flk1-GFP+ vessels (green) in the BM, showing vessels from leukemic mice have reduced diameter and increased distance from bone (arrows). Gray, bone collagen second harmonic generation (SHG).

(D) Representative 3D renderings of the surface of Flk1-GFP+ vessels (green) with the areas co-localizing with bone highlighted in pink. Dark blue in the background is bone.

(E) The contact area between vessels and bone is significantly reduced in AML-infiltrated BM. Data in (B)–(E) are from 5 control and 4 AML mice.

(F) Representative tile scan maximum projection (composite of individual tiles) of a Flk1-GFP mouse partially infiltrated with mTomato+ AML cells (red). Gray, bone collagen SHG. Time-lapse imaging shows steady and smooth vascular contours (red lines) in lightly infiltrated areas (P1). Instead, vessels in heavily infiltrated areas (P2) have irregular contours (red lines) and show active and inefficient sprouting (red arrows) over time.

(G) Selected frames from representative time-lapse data from a heavily infiltrated area showing rapid vascular sprout formation (red arrows) and regression (full time-lapse: Movie S1).

(H) VEGF-A levels in serum of control mice (C), mice with AML (AML), and mice with AML treated with combined cytarabine and doxorubicin (post-chemo). n = 4 mice per group. Error bars represent mean ± SEM.

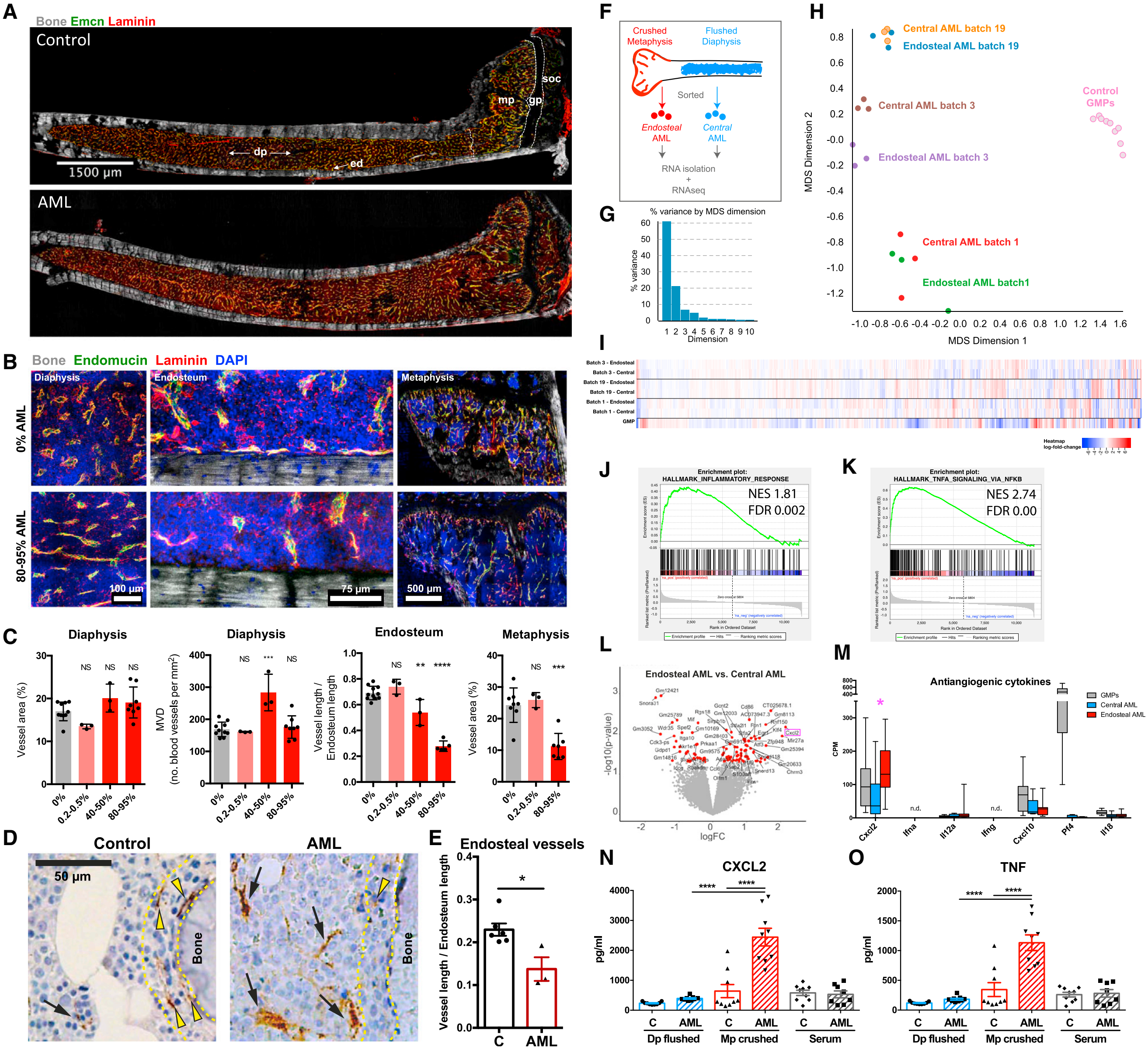

Figure 3. Endosteal and Metaphyseal Vessels Are Decreased in AML

(A) Representative maximum intensity projection of a tile scan (composite of individual tiles) of 20-μm-thick sections of undecalcified tibias from wild-type control (top) and fully infiltrated (bottom) mice. Vessels are labeled by laminin and endomucin (Emcn) immunostaining. dp, diaphysis; ed, endosteum; gp, growth plate; mp, metaphysis; soc, secondary ossification center.

(B) Representative maximum intensity projections comparing vascular staining in the diaphysis, endosteum, and metaphysis of control (top row) and fully infiltrated (bottom row) mice. Blue, DAPI; gray, bone collagen SHG; green, endomucin^{+} vessels; red, laminin^{+} vessels and extracellular matrix.

(C) Quantification of blood vessels in diaphysis, endosteum, and metaphysis at different stages of AML progression. Data were obtained from 11 mice with 0% infiltration (control), 3 mice with 0.2%–0.5% infiltration, 3 mice with 40%–50% infiltration, and 5–10 mice with 80%–95% infiltration from 2 independent cohorts. Error bars represent mean ± SD.

(D) Representative images of BM trephine biopsies from control and AML patients stained with anti-von Willebrand factor antibody to mark blood vessels (brown). Yellow dotted lines delineate endosteal area within 20 μm from the bone. Yellow arrowheads point at endosteal vessels and black arrowheads at central marrow vessels.

(E) Endosteal vessels are decreased in AML patients. Data were obtained from 6 control and 3 AML patients. Error bars represent mean ± SEM.

(F) Central and endosteal AML cells were isolated and analyzed by RNA-seq.

(G) Most of the variance in the data is explained by MDS dimensions 1 (60%) and 2 (21%).

(H) Multi-dimensional scaling (MDS) plot of distances between gene expression profiles of AML cells and control GMPs. Each dot represents a sample. Data were obtained from 3 AML batches, 3 biological replicates per batch, and 9 control mice.

(I) Heatmap of all the genes that are differentially regulated, with false detection rate (FDR) cutoff of 0.05. Gene expression is relative to GMP.

(legend continued on next page)

central AML cells originating from three independent primary donors to non-transformed granulocyte macrophage progenitors (GMPs) from the BM of healthy mice. Gene expression and multi-dimensional scale analyses illustrated that each AML batch had its own unique gene expression signature, consistent with clonal evolution of tumor cells, whereas control GMPs were extremely homogeneous (Figures 3G–3I).

Despite the similarity in gene expression between endosteal and central AML cells, gene set enrichment analysis (GSEA) demonstrated endosteal AML cells were enriched for expression of genes involved in the inflammatory response (Figure 3J) and tumor necrosis factor (TNF) signaling pathways (Figure 3K). Furthermore, the anti-angiogenic cytokine *Cxcl2* (also known as MIP-2α or chemokine gro-β; downstream of TNF; Tessier et al., 1997) was significantly more expressed in endosteal AML cells (Figures 3L and 3M). Analysis of TNF and CXCL2 levels in BM fluids confirmed that both cytokines were specifically and highly increased in endosteal areas of AML-burdened mice (Figures 3N and 3O). These results highlight the importance of inflammation in AML pathogenesis and support a role for CXCL2 and TNF in the remodeling of endosteal vessels.

BM Stroma Is Locally and Progressively Depleted in AML

Flow cytometry analysis (Figures S3C–S3H) demonstrated that, although the proportion of ECs in surviving stroma was increased in AML-burdened mice (Figure S3E), the absolute number of ECs was not statistically significantly different (Figure S3F). However, phenotypically defined endosteal ECs ($CD31^{hi}Endomucin^{hi}$ or $CD31^{+}Sca\text{-}1^{+}$) were significantly reduced in diseased mice (Figures S3G and S3H). To better understand how AML affects overall BM stroma, we imaged chimeric mice bearing membrane-bound $Tomato^{+}$ stroma and wild-type, non-fluorescent hematopoietic cells injected with yellow fluorescent protein $(YFP)^{+}GFP^{+}$ double-positive AML blasts. At >50% BM infiltration, we observed a dramatic reduction of overall stromal cells *in vivo*, including the stroma surrounding blood vessels and adjacent to bone (Figure S4A). Consistent with this pattern, extensive IVM time-lapse (7–12 hr) of these mice revealed that blood vessels underwent abnormal oscillations, suggesting that their anchorage to the surrounding parenchyma had been lost (Figure S4B; Movie S4, arrowheads). Extensive stroma loss was confirmed by flow cytometry analysis of non-chimeric mice, revealing a >10-fold reduction in the number of $CD45^{-}$ $Ter119^{-}$ cells in the BM of AML-burdened mice (Figure S4C). To better understand the process leading to such dramatic overhaul of BM stroma, we performed live imaging at earlier stages (days 10–12 post-injection of leukemia blasts; 5%–15% infiltration). At these earlier time points, we could compare areas with low and high infiltration within the same mouse (Figure S4D). Here, peri-vascular and endosteal stroma were depleted in highly infiltrated areas, whereas both components maintained a normal appearance in weakly infiltrated areas, suggesting that AML cells remodel the stroma locally after reaching a certain threshold density.

Loss of Healthy Hematopoiesis Is Temporally and Spatially Correlated with Endosteal Remodeling

Because the endosteal endothelium has been shown to locate next to and sustain osteoblasts (Kusumbe et al., 2014), we expanded our analysis of the endosteal microenvironment to include a focused investigation of osteoblastic cells (CFP^{+} or GFP^{+} cells in Col2.3-CFP/GFP reporter mice, respectively) during AML growth. IVM of the calvarium of Col2.3-GFP mice revealed that GFP^{+} osteoblastic cells were significantly reduced in an infiltration-dependent manner (Figures 4A and 4B). IVM of chimeras containing CFP^{+} osteoblastic cells, $mTomato^{+}$ healthy hematopoietic cells, and $YFP^{+}GFP^{+}$ leukemia revealed that AML cells, as they remodel stroma and vessels locally, also outcompete healthy hematopoietic cells and eliminate osteoblastic cells (Figures 4C and 4D). This finding indicated that microenvironmental and hematopoietic changes induced by leukemia evolve focally and in parallel. IVM of healthy and highly infiltrated double-transgenic Flk1-GFP/Col2.3-CFP mice confirmed that osteoblasts and endosteal vessels were lost in the presence of AML, whereas central vessels were maintained (Figure 4E). To understand whether one microenvironment component may be lost first, we analyzed long bone sections from mice at intermediate stages of disease (Figure 4F). Within the same bone, osteoblasts were significantly decreased only in areas with high levels of leukemic infiltration (Figure 4G), whereas we could detect loss of endosteal vessels in areas with intermediate levels of infiltration (Figure 4H). These data suggest that endosteal vessels may be lost earlier than osteoblasts.

We next investigated the hematopoietic changes associated with microenvironment remodeling. Analysis of non-chimeric mice with increasing AML infiltration by flow cytometry showed a progressive decrease of overall normal hematopoietic cells in the BM (Figure 1B) and more specifically of $Lineage^{-}$, $cKit^{+}$ $Sca\text{-}1^{+}$ (LKS) progenitor cell and LKS $CD48^{-}$ $CD150^{+}$ HSC populations (Figures 5A and S5A). Importantly, HSCs in the BM were significantly reduced only at late stages of disease (Figure 5A), when endosteal and metaphyseal vessels, as well as osteoblastic cells, were all drastically reduced (Figures 3 and 4). Furthermore, whereas LKS cells are lost in areas both distant from (flushed diaphysis) and close to (crushed metaphysis) the bone (Figure 5B), HSCs are most dramatically lost in the bone-rich metaphysis (Figure 5C). This observation highlights the association and consequent tandem collapse of endosteal vessels and HSCs at late stages of AML. We also observed that, as disease progresses, the number of HSCs in the spleen increases (Figures 5D, 5E, and S5B). Notably, this hematopoietic elevation coincides with an increase of splenic ECs (Figure 5F).

(J and K) Gene set enrichment analysis (GSEA) comparing AML cells isolated from central and endosteal BM areas for genes involved in (J) inflammatory response and in (K) the TNF signaling pathway.

(L) Volcano plot showing genes that are differentially expressed in endosteal and central AML cells. Red dots represent individual genes that are differentially expressed with a p value cutoff of 0.05. *Cxcl2* is highlighted and is overexpressed in endosteal AML cells.

(M) Expression of genes encoding cytokines known to inhibit angiogenesis.

(N and O) CXCL2 (N) and TNF (O) levels in central and endosteal BM fluid fractions and in the serum of the same mice. Data were obtained from 9 control and 9 AML-burdened mice.

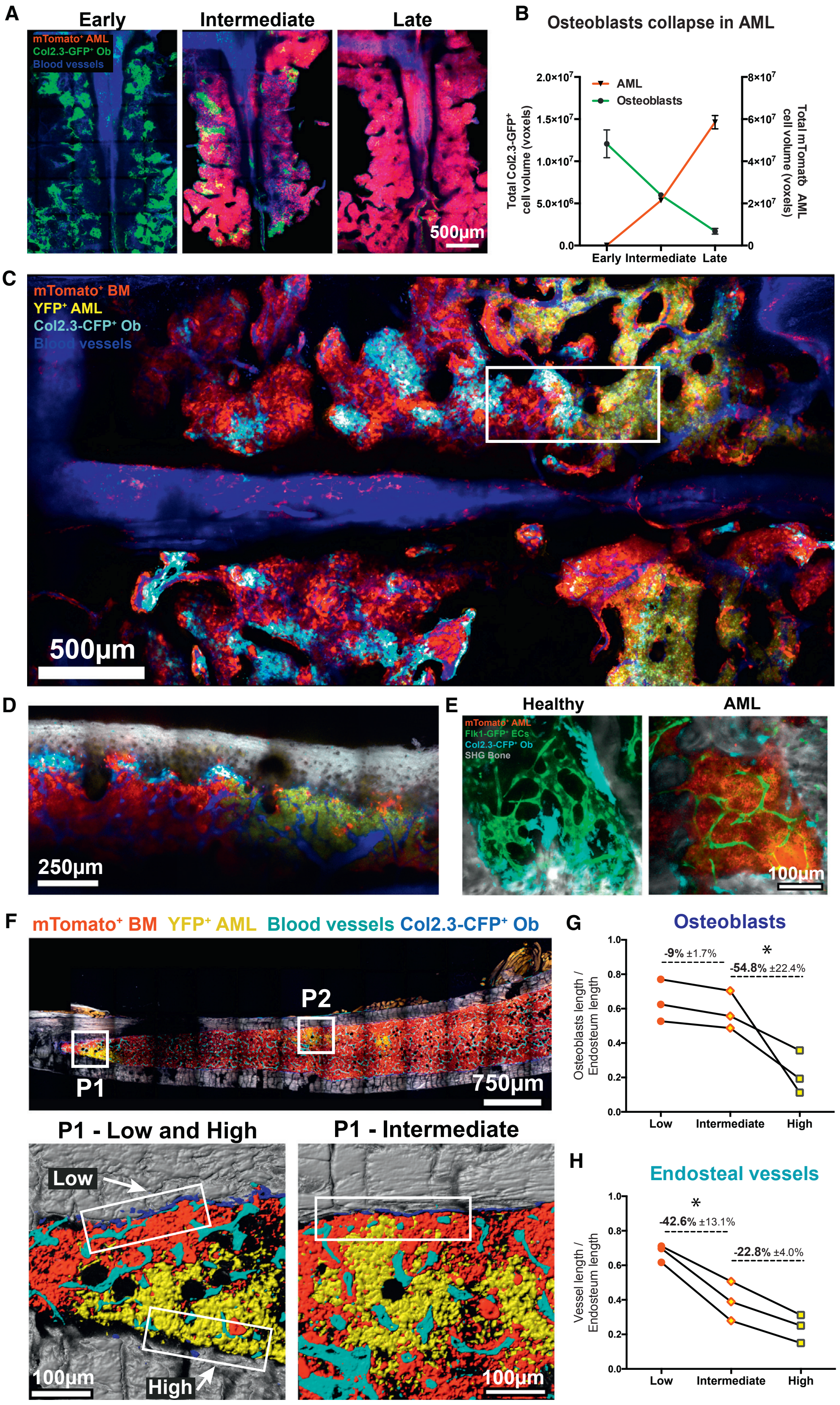

(legend on next page)

To reconcile our initial observations on the morphological and structural changes in BM vessels of AML-infiltrated animals (Figures 2 and 3) with the progressive loss of normal hematopoietic cells (Figures 5A–5F), we asked whether they could result in increased hematopoietic cell trafficking. We performed paired IVM of BM and spleen in the same AML-burdened and control mice (Figure S5C) and observed increased numbers of healthy hematopoietic cells adhering to (Figures 5G and 5H; Movie S5) and transmigrating across (Figures 5I and 5J; Movie S6) endothelial cells in leukemic mice. This pattern of egress may contribute to the loss of BM hematopoiesis. We also observed AML cell clusters that adhered to the endothelial cells on their intravascular surface and compromised blood flow (Movie S7), likely contributing to the observed functional alterations of the endothelium (Ramasamy et al., 2016).

AML-Induced Endosteal Remodeling Regulates HSC Numbers

To confirm that HSC loss from the BM was due to the microenvironmental changes we observed rather than a direct effect of leukemia cells on the stem cells, we asked whether the remodeled BM would still have the capacity to support homing of HSCs. To this end, we transplanted DiD-labeled HSCs into lethally irradiated control and leukemic mice (Figure 6A). Two days after transplantation, significantly lower numbers of HSCs were found in the BM of leukemic mice (Figure 6B). This observation suggests that AML leads to a specific collapse of HSC-supportive BM niches, as previously hypothesized for B cell acute lymphoblastic leukemia (Colmone et al., 2008). We then investigated whether maintenance of BM endosteal endothelium during AML growth would protect HSCs in endosteal areas. To address this, we treated leukemic mice with deferoxamine (DFO), a clinically approved prolyl-4-hydroxylase (PHD) inhibitor normally administered as an iron chelator but also recently described to induce endosteal vessel expansion through enhancement of hypoxia-inducible factor 1α (Hif-1α) stability and activity (Kusumbe et al., 2014). DFO or control (PBS) treatment started 8 days post-injection of AML blasts and continued until day 22 post-transplantation, at which time point the BM was heavily infiltrated (Figure 6C). DFO-treated mice had similar numbers of AML cells in the BM (Figure 6D) and similar disease progression (Figures S6A and S6B) and survival (Figure S6C). Endosteal blood vessels were increased in DFO-treated mice (Figures 6E and 6F). Consistent with our hypothesis that HSC numbers depend on endosteal vessels, we observed that leukemic mice receiving DFO had significantly higher numbers of HSCs in the trabecular-rich metaphysis, but not in flushed diaphyseal BM (Figures 6G and 6H). A direct positive effect of DFO on HSC numbers was excluded through *in vitro* culture (Figures S6D–S6G). To further investigate the clinical utility of DFO, we investigated the homing of HSCs in mice infiltrated with AML and treated with DFO or vehicle (Figure 6I). In line with our hypothesis, DFO-treated mice supported HSC homing to the BM (Figure 6J). Altogether, these data suggested that rescue of endosteal vessels can support HSCs despite AML growth and improve HSC homing.

Rescue of Endosteal Vessels Improves Chemotherapy Efficiency

The observed remodeling of blood vessels in AML-burdened mice and especially the loss of endosteal vessels led us to hypothesize that these changes could not only contribute to outcompeting healthy hematopoiesis but also compromise delivery of chemotherapy. In a xenograft transplantation model of AML, chemo-resistant leukemia cells were previously shown to locate near the endosteum (Ishikawa et al., 2007). This suggests that localization of leukemia cells in areas of BM stripped of their vasculature could provide them with a survival advantage. In our model, trabecular areas were enriched for AML cells both at early and late stages of infiltration and following induction chemotherapy (Figure S7A). We hypothesized that, by rescuing blood vessels in endosteal areas, we could increase chemotherapy delivery and therefore efficacy. To test this prediction, we utilized Fbxw7$^{i\Delta EC}$ mutant mice, in which tamoxifen administration leads to increased activation of Notch signaling specifically in ECs, thereby increasing the number of endosteal vessels and arterioles (Kusumbe et al., 2016; Ramasamy et al., 2014). Fully infiltrated Fbxw7$^{i\Delta EC}$ mutants had increased numbers of endosteal vessels (Figures 7A and 7B). At this point, we treated both Fbxw7$^{i\Delta EC}$ and control mice with an adapted form of clinical induction chemotherapy (cytarabine [Ara-C] and doxorubicin [Doxo]; Wunderlich et al., 2013; Figures 7C and S7B–S7D). In agreement with a previous report (Hooper et al., 2009), we observed significant chemotherapy-induced damage to the BM vasculature, including endosteal vessels, in both control and mutant mice (Figure 7D). We observed that, after treatment, the Fbxw7$^{i\Delta EC}$ mutants had reduced numbers of surviving AML

Figure 4. AML Remodels the Endosteal Niche and Outcompetes Normal Hematopoiesis

(A) Representative tile scans (composite of individual tiles) of Col2.3-GFP mice transplanted with mTomato$^+$ leukemia. Blue, Cy5-dextran$^+$ blood vessels; green, Col2.3-GFP$^+$ osteoblastic cells; red, mTomato$^+$ AML.

(B) Automated segmentation and volume calculation (voxels) of osteoblast loss (green line) and leukemia expansion (red line) over time. Data were obtained from 17 mice, from 2 independent cohorts. Error bars represent mean ± SEM.

(C) Maximum intensity projection of a tile scan (composite of individual tiles) of a Col2.3-CFP recipient with mTomato$^+$ healthy hematopoietic cells and YFP$^+$GFP$^+$ AML blasts. Leukemia cells (yellow) infiltrate the calvarium and deplete osteoblastic cells (cyan) and healthy hematopoietic cells (red) locally.

(D) 2D slice from the area framed in (C) at a depth close to the calvarium surface, including the BM components from (C) and collagen bone SHG (gray). (C and D) Data were obtained from 6 mice.

(E) IVM images of representative areas of the calvarium BM of control and AML-infiltrated Col2.3-CFP/Flk1-GFP double-transgenic mice. Cyan, Col2.3-GFP$^+$ osteoblastic cells; gray, bone; green, Flk1-GFP$^+$ ECs; red, mTomato$^+$ AML cells. n = 3 mice.

(F) Maximum intensity projection of a representative tile scan (composite of individual tiles) of undecalcified tibia sections from Col2.3-CFP recipients with mTomato$^+$ healthy hematopoietic cells (red) and YFP$^+$GFP$^+$ AML (yellow) blasts. Blue, osteoblasts; cyan, vessels (endomucin$^+$); gray, bone. Mice had intermediate levels of AML infiltration. Boxes in P1 and P2 higher magnification images illustrate examples of areas within the same bone with low, high, and intermediate levels of infiltration, used to quantify stroma remodeling.

(G and H) Quantification of osteoblasts (G) and endosteal vessels (H). Data were obtained from 3 mice.

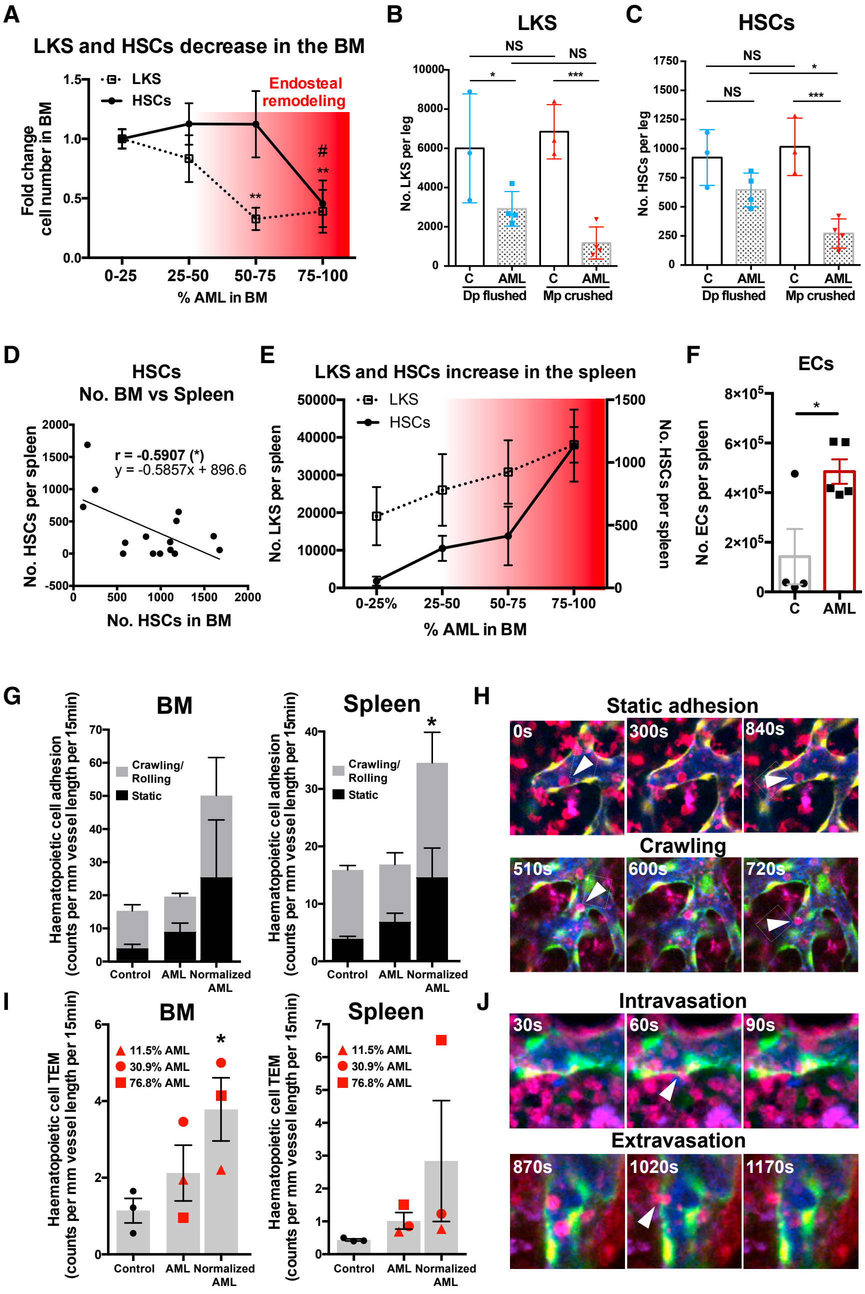

Figure 5. HSC Dynamics and Hematopoietic Cell Trafficking in BM and Spleen

(A) Fold change in LKS cells and HSCs with increasing AML infiltration. HSCs are only lost at late time points, when endosteal remodeling is more dramatic. Data were obtained from 15 mice with 0%–25%, 5 mice with 25%–50%, 6 mice with 50%–75%, and 8 mice with 75%–100% AML infiltration, from 2 independent cohorts. Error bars represent mean ± SEM.

(B) LKS cells are significantly decreased in both the diaphysis (Dp flushed) and in the trabecular bone-rich metaphysis (Mp crushed) of AML-burdened mice, with no differences between these two fractions.

(C) HSCs are significantly lost in the metaphysis of AML-burdened mice. Data were obtained from 3 control and 4 leukemic mice. Error bars represent mean ± SD.

(D) Paired analysis shows a negative correlation between HSC numbers in spleen and BM (1 femur, 2 tibias, and 2 ileac bones).

(E) LKS cells and HSC numbers increase in the spleen during AML progression. Data were obtained from 7 mice with 0%–25%, 3 mice with 25%–50%, 2 mice with 50%–75%, and 3 mice with 75%–100% BM infiltration. Error bars represent mean ± SEM.

(F) Mice burdened with AML have significantly increased absolute cell numbers of $CD31^{+}$ ECs in the spleen. Data were obtained from 4 control and 5 leukemic mice. Error bars represent mean ± SEM.

(G) Time-lapse *in vivo* imaging revealed that, in AML-burdened mice, residual healthy $mTomato^{+}$ cells had increased adhesion to the luminal endothelial surface, particularly in leukemic spleens, when normalized by the frequency of surviving cells (e.g., once divided by 0.699 in a mouse with 30.1% blasts in the BM).

(H) Examples of healthy $tomato^{+}$ cells maintaining stable (static) or transient (crawling) adhesion to the splenic endothelium.

(I) We observed a significant increase of healthy mTomato+ cells undergoing transendothelial migration (TEM) in the BM of AML-burdened mice, once normalized for the infiltration level.

(J) Examples of cells migrating from the tissue to the vascular lumen (intravasation) and in the opposite direction (extravasation) are shown.

(C and E) Blue, Cy5-dextran; green, Flk1-GFP^{+} ECs; red, $mTomato^{+}$ healthy hematopoietic cells. Data were obtained from the analysis of 521 (BM) and 588 (spleen) $tomato^{+}$ cells from 3 control and 3 leukemic mice. Error bars represent mean ± SEM.

cells in the BM (Figure 7E), delayed relapse (Figure 7F), and increased survival (Figure 7G). Altogether, these data suggest that the rescue of endosteal vessels before induction chemotherapy can improve its efficacy.

DISCUSSION

Our study describes how AML cells focally and progressively remodel BM vasculature to transform HSC niches into preferential leukemia microenvironments. In particular, BM vasculature editing is anatomically diverse, with endosteal vessels being progressively obliterated, whereas central marrow vessels survive, albeit with compromised function. The two main consequences of this are HSC loss specifically from endosteal areas and survival of leukemia cells following chemotherapy treatment. Intravital microscopy provided us with the unique opportunity to perform detailed, longitudinal analysis of progressive changes occurring across the entire vascular bed irrigating the calvarium BM. This approach identified dynamic and area-specific changes previously overlooked by studies of AML patient

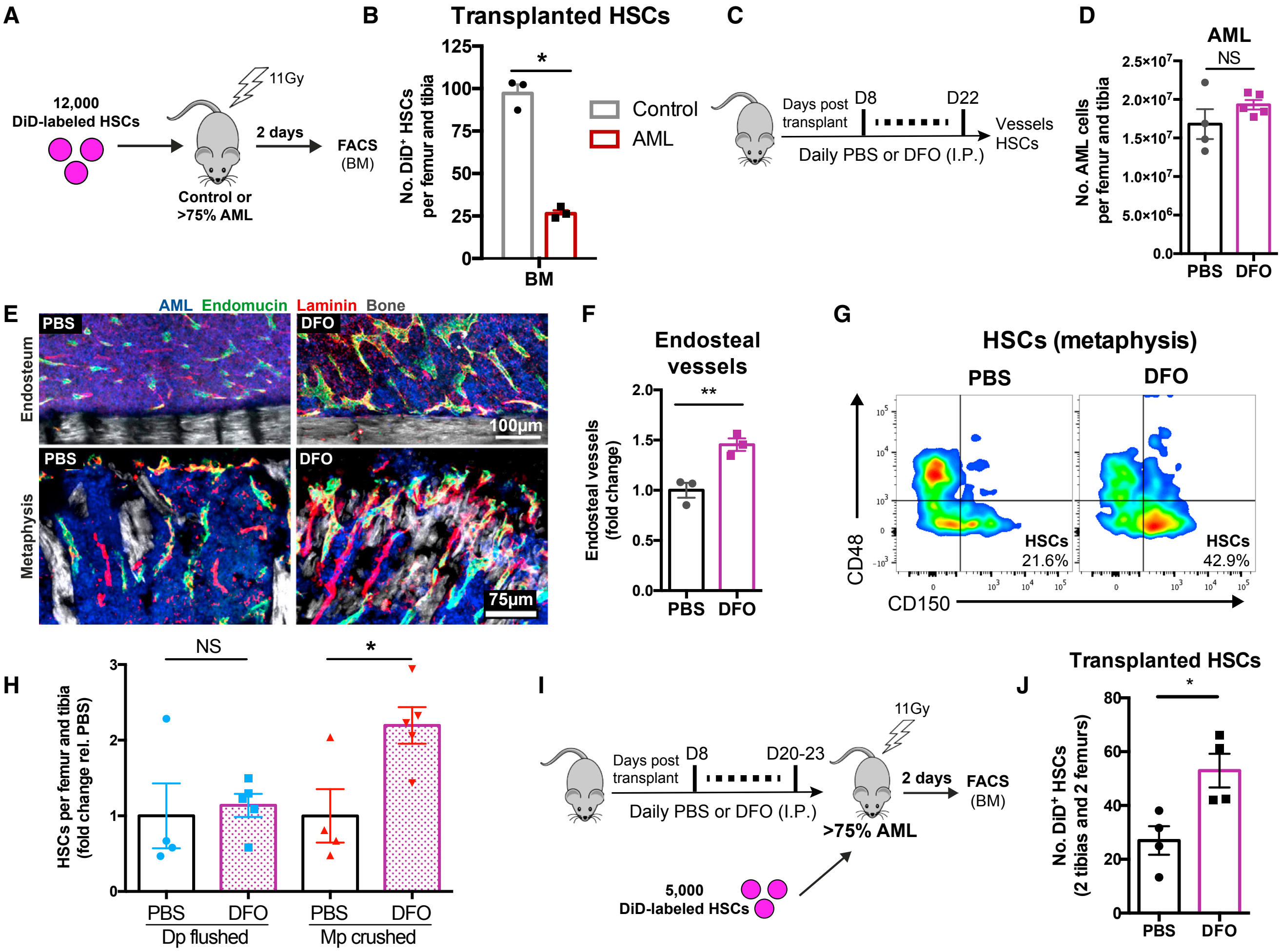

Figure 6. The Endosteal Remodeling in AML Regulates HSC Numbers

(A) 12,000 sort-purified HSCs were labeled with DiD and transplanted into irradiated control or AML-recipient mice. 2 days after transplantation, DiD^{+} HSCs resident in the BM were quantified by flow cytometry.

(B) Homing of transplanted DiD^{+} HSCs was significantly impaired in the BM of AML-burdened mice. n = 3 control and 3 leukemic mice.

(C) DFO treatment regimen.

(D–F) DFO did not affect the total number of AML cells (D) but increased the number of endosteal vessels (E and F). Blue, AML cells; gray, collage bone SHG; green, $endomucin^{+}$ vessels; red, $laminin^{+}$ vessels and extracellular matrix. Each dot is a mouse.

(G) AML-burdened mice treated with DFO had more HSCs remaining in the metaphysis in comparison to controls.

(H) Quantification of flow cytometry analysis of DFO treated and control (PBS) mice, showing similar numbers of HSCs in the diaphysis (Dp flushed) but a significant increase of HSCs remaining in the metaphysis (Mp crushed).

Data are representative of (G) and obtained from (H) 4 mice treated with PBS and 5 treated with DFO.

(I) Mice transplanted with AML cells were treated with either PBS or DFO. At full infiltration, mice were lethally irradiated and transplanted with DiD-labeled HSCs that had not been exposed to DFO.

(J) DFO improves the homing of transplanted DiD^{+} HSCs in the BM of AML-burdened mice. Data were obtained from 4 recipients treated with PBS and 4 recipients treated with DFO.

In (B–J), error bars represent mean ± SEM.

samples that focused on central BM areas or examined vasculature as a whole (Aguayo et al., 2000; Hanoun et al., 2014; Hussong et al., 2000; Padró et al., 2000). Consistent with initial reports of the angiogenic potential of AML cells (Fiedler et al., 1997), we observed increased levels of VEGF-A in leukemic mice and endothelial cell dynamics resembling angiogenic sprouting (Gerhardt et al., 2003). However, these sprouts never gave rise to the formation of new vessels, which might be driven by VEGF-A-mediated tip cell competition (Jakobsson et al., 2010). The expression of additional angiogenic factors by AML cells might also explain the disappointing results of the clinical trials testing anti-angiogenic therapies for AML patients (Fiedler et al., 2003; Ossenkoppele et al., 2012; Zahiragic et al., 2007). Furthermore, the combination of high-resolution intravital microscopy and quantitative flow cytometry analysis of BM ECs showed that the apparent relative increase in ECs is a consequence of the overall loss of BM stroma. This finding contrasts with observations made in solid tumors, where most

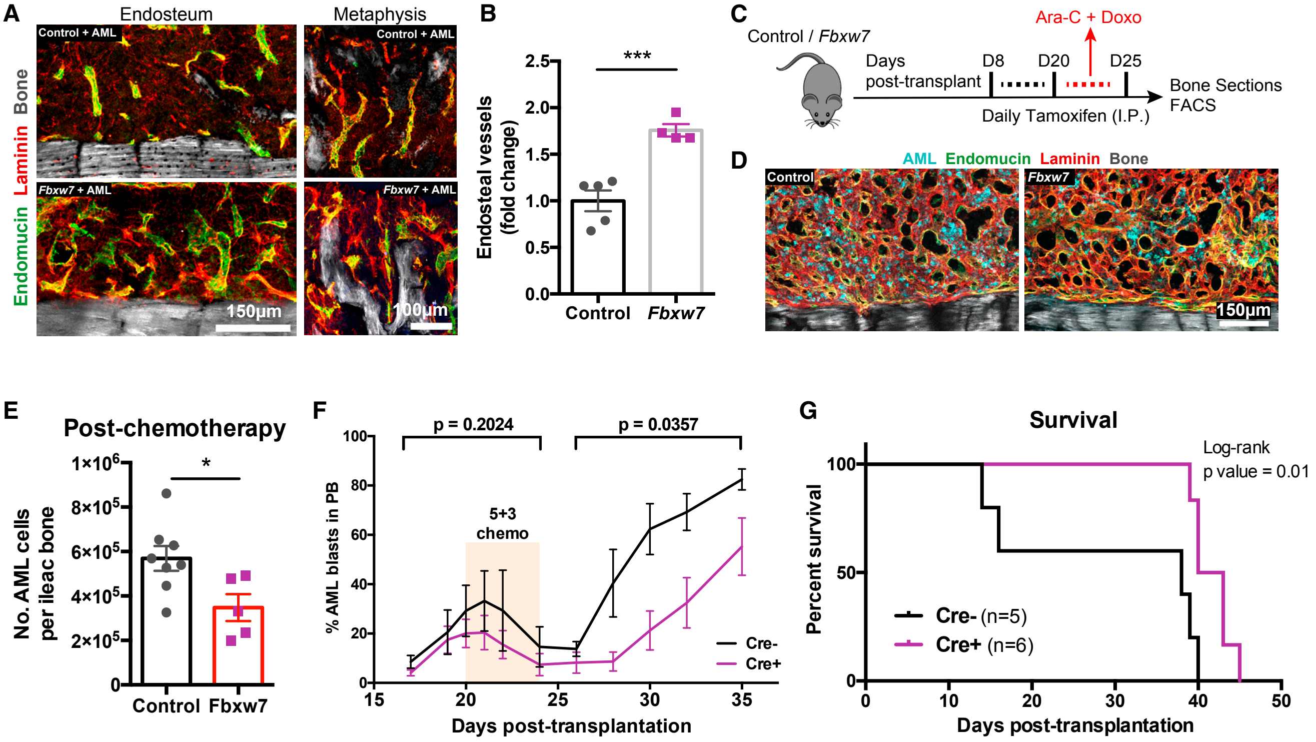

Figure 7. Rescue of Endosteal Vessels Increases Induction Chemotherapy Efficiency

(A) Immunofluorescence staining of endosteal areas showing a significant increase of blood vessels in Fbxw7$^{i\Delta EC}$ mutants infiltrated with AML. Gray, bone collagen SHG; green, endomucin^{+} vessels; red, laminin^{+} vessels and extracellular matrix.

(B) Quantification of the results obtained from 5 control mice and 4 Fbxw7$^{i\Delta EC}$ mice.

(C) Scheme of treatment regimen used to delete Fbxw7 in ECs.

(D) Maximum projections of immunofluorescence staining of representative endosteal areas showing dilated blood vessels (green, endomucin; red, laminin) after therapy in both control and mutant animals, as well as surviving AML cells (cyan) scattered through the tissue. Data are representative of 3 control mice and 3 Fbxw7$^{i\Delta EC}$ mutants.

(E) After chemotherapy, there was a significant decrease of surviving AML cells in Fbxw7$^{i\Delta EC}$ mutants, where the endosteal vessels had been rescued. Data were obtained from 8 control mice and 5 Fbxw7$^{i\Delta EC}$ mutants.

(F) Although disease progression before chemotherapy is similar, relapse is delayed in Fbxw7$^{i\Delta EC}$ mutants. n = 3 Cre^{-} and 6 Cre^{+} mice.

(G) Kaplan-Meyer curve showing improved survival in treated Fbxw7$^{i\Delta EC}$ mutants transplanted with AML. n = 5 Cre^{-} and 6 Cre^{+} mice.

In (B–F), error bars represent mean ± SEM.

often accumulation of stroma and pro-fibrotic changes are reported adjacent to the cancerous cells (Quail and Joyce, 2013).

Whereas central marrow vessels were maintained during AML infiltration, we observed dramatic remodeling of endosteal vessels. These vessels were initially remodeled in areas containing foci of disease but eventually almost disappeared from the BM of heavily infiltrated animals. AML cells in endosteal areas were enriched in inflammatory and TNF gene signatures and expressed higher levels of CXCL2. Both TNF and CXCL2 levels were locally increased in endosteal areas. This is consistent with the recently described role of TNF in vascular destruction (Kammertoens et al., 2017). Furthermore, CXCL2 is an angiogenesis inhibitor (Cao et al., 1995) that has also been shown to mobilize HSCs from the BM (Fukuda et al., 2007) and to be associated with poor prognosis and reduced survival in AML (Katsumura et al., 2016). Overall, our data support future investigation of the role of CXCL2 and TNF in the vascular remodeling and loss of HSCs induced by AML in endosteal regions.

In parallel with endosteal vessel remodeling, we also observed a similar pattern of osteoblastic cell loss. It was shown previously in a mouse model of myeloproliferative neoplasia based on widespread induction of BCR-ABL that aberrant differentiation of mesenchymal progenitor cells led to an expansion of dysfunctional osteoblasts associated with loss of HSC activity (Schepers et al., 2013). Here, we observed the depletion of not only osteoblasts but also endosteal vessels. This interpretation does not exclude "re-wiring" of mesenchymal progenitors; however, it highlights a dramatic imbalance between osteoblast generation and loss. Most importantly, our findings are consistent with studies that reported decreased numbers and activity of osteoblasts in patients with AML (Krevvata et al., 2014). Using a complementary experimental model, Hanoun and colleagues have shown that sympathetic neuropathy promotes the expansion of nestin^{+} mesenchymal stem cells and limits their differentiation into NG2^{+} cells in the arteriolar niche of mice with AML (Hanoun et al., 2014). These data, when combined with our results presented here, suggest that fast-growing AML depletes stroma with niche function.

By combining longitudinal intravital microscopy and immunofluorescence analyses with the study of mice with intermediate

disease burden, we obtained detailed temporal information on the progression of AML-induced BM remodeling. We have recently reported a similar collapse of the osteolineage at late stages of T-ALL (Hawkins et al., 2016). In contrast with AML, here we observed that endosteal vessels are not lost in T-ALL, highlighting the specificity of microenvironment remodeling in different types of leukemia. However, analysis of human AML samples revealed endosteal vessel remodeling in unrelated AML types. Our findings, together with clinical evidence demonstrating that cytopenias are more common and severe in AML than in T-ALL, suggests that endosteal vessel remodeling may be the cause of dramatic cytopenias associated with AML.

Splenomegaly, though rare in AML patients, is present and accompanied by extramedullary hematopoiesis in experimental mouse models of acute leukemia. In our model, increased EC numbers in the spleen likely form *de novo* vascular niches able to support HSCs and extramedullary hematopoiesis, consistent with the recent discovery of a peri-sinusoidal niche in the spleen (Inra et al., 2015). Moreover, we were able to detect functional changes in endothelial cells, allowing for greater adhesion and transendothelial migration of hematopoietic cells in diseased mice. This is consistent with a recent study highlighting increased vascular permeability in AML (Passaro et al., 2017). These changes likely contribute to the egress of healthy hematopoietic cells from the BM (Bixel et al., 2017; Itkin et al., 2016) and relocation to the spleen.

Consistent with previous studies indicating that HSCs are relatively resistant to AML invasion (Cheng et al., 2015; Miraki-Moud et al., 2013), we observed that HSC loss occurs at late stages of disease infiltration, when we show that endosteal remodeling is more evident. Treatment with DFO significantly increased the number of endosteal blood vessels together with endosteal HSCs and improved the homing of HSCs to the BM. DFO has been reported to limit leukemic cell proliferation and to potentiate the effect of differentiation therapies in AML through iron chelation (Callens et al., 2010). Here, no significant changes in the number of AML cells or disease progression were observed in DFO-treated mice. This suggests that potential off-target effects of DFO on AML proliferation did not play a major role in the protection of HSCs.

Apart from leading to HSC loss, elimination of endosteal vessels may favor AML cells during chemotherapy administration by providing them with a vessel-poor microenvironment where drug delivery would likely be challenged and/or inefficient and therefore would foster chemo-resistance. Consistent with our hypothesis, chemo-resistant AML has been shown to initiate relapse from endosteal areas (Ishikawa et al., 2007). Our genetic approach showed that endosteal vessels can be rescued in AML-burdened animals and, in doing so, the efficacy of chemotherapy treatment increases. Our study suggests that induction of endosteal vessels and vascular normalization are promising avenues to both safeguard residual healthy hematopoiesis and improve chemotherapy treatment of AML patients.

STAR★METHODS

Detailed methods are provided in the online version of this paper and include the following:

- KEY RESOURCES TABLE
- CONTACT FOR REAGENT AND RESOURCE SHARING
- EXPERIMENTAL MODEL AND SUBJECT DETAILS
  - Mice
  - AML experimental model
  - T-ALL experimental model
  - Human samples
- METHOD DETAILS
  - Drug treatments
  - BM chimeras
  - Flow cytometry
  - HSC transplantation and homing analysis
  - LKS culture and DFO *in vitro* treatment
  - RNA sequencing and analysis
  - Enzyme-linked immunosorbent assay (ELISA)
  - Intravital microscopy
  - Immunofluorescence of undecalcified long bone sections
  - Human trephine biopsies
  - Image processing and quantification
- QUANTITATION AND STATISTICAL ANALYSES
- DATA AND SOFTWARE AVAILABILITY

SUPPLEMENTAL INFORMATION

Supplemental Information includes seven figures and seven movies and can be found with this article online at https://doi.org/10.1016/j.stem.2017.11.006.

ACKNOWLEDGMENTS

D.D. was supported by the GABBA PhD program (FCT fellowship SFRH/BD/52195/2013) and by the European Haematology Association (EHA)-American Society of Hematology (ASH) Translational Research Training in Hematology (TRTH) program; C.L.C., by Bloodwise (12033), HFSP (RGP0051/2011), CRUK (C36195/A1183), BBSRC (BB/I004033/1), KKLF (KKL460), and European Research Council (ERC) (337066); E.D.H., by the EHA and Bloodwise (12033); K.D.F., by the Wellcome Trust (201356/Z/16/Z); L.M.C., by the Medical Research Council (MR/M01245X/1), the NHLI Foundation, and core funding from Cancer Research UK; and R.H.A., by ERC (AdG 339409 AngioBone). S.K.R. is a Sir Henry Dale Fellow jointly supported by the Wellcome Trust and the Royal Society (202300/Z/16/Z). A.P.K. is an MRC fellow (MR/PO2209X/1). S.J.V. was funded by a Rubicon fellowship from the Netherlands Organization for Scientific Research. We are grateful to A. Medvinsky (U. of Edinburgh) for his kind gift of Flk1-GFP mice and to C. Nerlov for the Pu1-YFP mice. We thank D. Keller, S. Rothery (Imperial College FILM facility), and N. Sergent (Carl Zeiss) for support with microscopy; S. Piperelis, E. Ibarguen, W. Steel, H. Goyal, and C. Godfrey (Imperial College CBS facility) for logistical help; J. Srivastava, C. Simpson, and J. Rowley for support from the flow cytometry facility; Arwa Kocache (Imperial College Healthcare NHS Trust) for supplying cytarabine and doxorubicin; and R.E. Sinden, H. Fleming, K. Hodivala-Dilke, and R.W. Johnstone for critical reading of the manuscript.

AUTHOR CONTRIBUTIONS

D.D. and C.L.C. conceived the project. D.D., E.D.H., and L.E.P. refined bone immunofluorescence methods. D.D., E.D.H., O.A., K.D.F., L.M.C., and C.L.C. established imaging platforms and performed imaging experiments. D.D., E.D.H., H.A., M.H., C.P., and N.R. conducted animal and flow cytometry experiments. I.Y.K., S.J.V., E.D.H., and D.D. performed RNA-seq experiments. L.S., C.M., A.W., and L.E.P. provided and analyzed human samples. K.D.F. and D.D. performed ELISA experiments. T.S.W. and K.D. performed mathematical analysis. D.D., K.D.F., R.K., L.S., and C.P. performed image analysis. S.K.R., A.P.K., and R.H.A. contributed to experimental designing and provided

CellPress

the Fbxw7 mutants. D.D. and C.L.C. analyzed data and wrote the manuscript. All authors provided critical feedback on the manuscript.

Received: April 6, 2017
Revised: September 15, 2017
Accepted: November 6, 2017
Published: December 21, 2017

REFERENCES

Aguayo, A., Kantarjian, H., Manshouri, T., Gidel, C., Estey, E., Thomas, D., Koller, C., Estrov, Z., O'Brien, S., Keating, M., et al. (2000). Angiogenesis in acute and chronic leukemias and myelodysplastic syndromes. Blood *96*, 2240–2245.

Aster, J.C., Xu, L., Karnell, F.G., Patriub, V., Pui, J.C., and Pear, W.S. (2000). Essential Roles for Ankyrin Repeat and Transactivation Domains in Induction of T-Cell Leukemia by Notch1. Mol. Cell. Biol *20*, 7505–7515.

Bixel, M.G., Kusumbe, A.P., Ramasamy, S.K., Sivaraj, K.K., Butz, S., Vestweber, D., and Adams, R.H. (2017). Flow dynamics and HSPC homing in bone marrow microvessels. Cell Rep. *18*, 1804–1816.

Callens, C., Coulon, S., Naudin, J., Radford-Weiss, I., Boissel, N., Raffoux, E., Wang, P.H., Agarwal, S., Tamouza, H., Paubelle, E., et al. (2010). Targeting iron homeostasis induces cellular differentiation and synergizes with differentiating agents in acute myeloid leukemia. J. Exp. Med. *207*, 731–750.

Cao, Y., Chen, C., Weatherbee, J.A., Tsang, M., and Folkman, J. (1995). gro-beta, a -C-X-C- chemokine, is an angiogenesis inhibitor that suppresses the growth of Lewis lung carcinoma in mice. J. Exp. Med. *182*, 2069–2077.

Cheng, H., Hao, S., Liu, Y., Pang, Y., Ma, S., Dong, F., Xu, J., Zheng, G., Li, S., Yuan, W., and Cheng, T. (2015). Leukemic marrow infiltration reveals a novel role for Egr3 as a potent inhibitor of normal hematopoietic stem cell proliferation. Blood *126*, 1302–1313.

Colmone, A., Amorim, M., Pontier, A.L., Wang, S., Jablonski, E., and Sipkins, D.A. (2008). Leukemic cells create bone marrow niches that disrupt the behavior of normal hematopoietic progenitor cells. Science *322*, 1861–1865.

Döhner, H., Weisdorf, D.J., and Bloomfield, C.D. (2015). Acute myeloid leukemia. N. Engl. J. Med. *373*, 1136–1152.

Duan, C.W., Shi, J., Chen, J., Wang, B., Yu, Y.H., Qin, X., Zhou, X.C., Cai, Y.J., Li, Z.Q., Zhang, F., et al. (2014). Leukemia propagating cells rebuild an evolving niche in response to therapy. Cancer Cell *25*, 778–793.

Dunne, J., Gascoyne, D.M., Lister, T.A., Brady, H.J., Heidenreich, O., and Young, B.D. (2010). AML1/ETO proteins control POU4F1/BRN3A expression and function in t(8;21) acute myeloid leukemia. Cancer Res *70*, 3985–3995.

Fiedler, W., Graeven, U., Ergün, S., Verago, S., Kilic, N., Stockschläder, M., and Hossfeld, D.K. (1997). Vascular endothelial growth factor, a possible paracrine growth factor in human acute myeloid leukemia. Blood *89*, 1870–1875.

Fiedler, W., Mesters, R., Tinnefeld, H., Loges, S., Staib, P., Duhrsen, U., Flasshove, M., Ottmann, O.G., Jung, W., Cavalli, F., et al. (2003). A phase 2 clinical study of SU5416 in patients with refractory acute myeloid leukemia. Blood *102*, 2763–2767.

Fukuda, S., Bian, H., King, A.G., and Pelus, L.M. (2007). The chemokine GRObeta mobilizes early hematopoietic stem cells characterized by enhanced homing and engraftment. Blood *110*, 860–869.

Gerhardt, H., Golding, M., Fruttiger, M., Ruhrberg, C., Lundkvist, A., Abramsson, A., Jeltsch, M., Mitchell, C., Alitalo, K., Shima, D., and Betsholtz, C. (2003). VEGF guides angiogenic sprouting utilizing endothelial tip cell filopodia. J. Cell Biol. *161*, 1163–1177.

Hannon, G.J., Sun, P., Carnero, A., Xie, L.Y., Maestro, R., Conklin, D.S., and Beach, D. (1999). MaRX: an approach to genetics in mammalian cells. Science *283*, 1129–1130.

Hanoun, M., Zhang, D., Mizoguchi, T., Pinho, S., Pierce, H., Kunisaki, Y., Lacombe, J., Armstrong, S.A., Dührsen, U., and Frenette, P.S. (2014). Acute myelogenous leukemia-induced sympathetic neuropathy promotes malignancy in an altered hematopoietic stem cell niche. Cell Stem Cell *15*, 365–375.

Hawkins, E.D., Hommel, M., Turner, M.L., Battye, F.L., Markham, J.F., and Hodgkin, P.D. (2007). Measuring lymphocyte proliferation, survival and differentiation using CFSE time-series data. Nat. Protoc. *2*, 2057–2067.

Hawkins, E.D., Oliaro, J., Ramsbottom, K.M., Ting, S.B., Sacirbegovic, F., Harvey, M., Kinwell, T., Ghysdael, J., Johnstone, R.W., Humbert, P.O., and Russell, S.M. (2014). Lethal giant larvae 1 tumour suppressor activity is not conserved in models of mammalian T and B cell leukaemia. PLoS ONE *9*, e87376.

Hawkins, E.D., Duarte, D., Akinduro, O., Khorshed, R.A., Passaro, D., Nowicka, M., Straszkowski, L., Scott, M.K., Rothery, S., Ruivo, N., et al. (2016). T-cell acute leukaemia exhibits dynamic interactions with bone marrow microenvironments. Nature *538*, 518–522.

Headley, M.B., Bins, A., Nip, A., Roberts, E.W., Looney, M.R., Gerard, A., and Krummel, M.F. (2016). Visualization of immediate immune responses to pioneer metastatic cells in the lung. Nature *531*, 513–517.

Hérault, A., Binnewies, M., Leong, S., Calero-Nieto, F.J., Zhang, S.Y., Kang, Y.A., Wang, X., Pietras, E.M., Chu, S.H., Barry-Holson, K., et al. (2017). Myeloid progenitor cluster formation drives emergency and leukaemic myelopoiesis. Nature *544*, 53–58.

Hooper, A.T., Butler, J.M., Nolan, D.J., Kranz, A., Iida, K., Kobayashi, M., Kopp, H.G., Shido, K., Petit, I., Yanger, K., et al. (2009). Engraftment and reconstitution of hematopoiesis is dependent on VEGFR2-mediated regeneration of sinusoidal endothelial cells. Cell Stem Cell *4*, 263–274.

Hussong, J.W., Rodgers, G.M., and Shami, P.J. (2000). Evidence of increased angiogenesis in patients with acute myeloid leukemia. Blood *95*, 309–313.

Inra, C.N., Zhou, B.O., Acar, M., Murphy, M.M., Richardson, J., Zhao, Z., and Morrison, S.J. (2015). A perisinusoidal niche for extramedullary haematopoiesis in the spleen. Nature *527*, 466–471.

Ishikawa, F., Yoshida, S., Saito, Y., Hijikata, A., Kitamura, H., Tanaka, S., Nakamura, R., Tanaka, T., Tomiyama, H., Saito, N., et al. (2007). Chemotherapy-resistant human AML stem cells home to and engraft within the bone-marrow endosteal region. Nat. Biotechnol. *25*, 1315–1321.

Itkin, T., Gur-Cohen, S., Spencer, J.A., Schajnovitz, A., Ramasamy, S.K., Kusumbe, A.P., Ledergor, G., Jung, Y., Milo, I., Poulos, M.G., et al. (2016). Distinct bone marrow blood vessels differentially regulate haematopoiesis. Nature *532*, 323–328.

Jakobsson, L., Franco, C.A., Bentley, K., Collins, R.T., Ponsioen, B., Aspalter, I.M., Rosewell, I., Busse, M., Thurston, G., Medvinsky, A., et al. (2010). Endothelial cells dynamically compete for the tip cell position during angiogenic sprouting. Nat. Cell Biol. *12*, 943–953.

Kammertoens, T., Friese, C., Arina, A., Idel, C., Briesemeister, D., Rothe, M., Ivanov, A., Szymborska, A., Patone, G., Kunz, S., et al. (2017). Tumour ischaemia by interferon-γ resembles physiological blood vessel regression. Nature *545*, 98–102.

Katsumura, K.R., Ong, I.M., DeVilbiss, A.W., Sanalkumar, R., and Bresnick, E.H. (2016). GATA factor-dependent positive-feedback circuit in acute myeloid leukemia cells. Cell Rep. *16*, 2428–2441.

Khorshed, R.A., Hawkins, E.D., Duarte, D., Scott, M.K., Akinduro, O.A., Rashidi, N.M., Spitaler, M., and Lo Celso, C. (2015). Automated identification and localization of hematopoietic stem cells in 3D intravital microscopy data. Stem Cell Reports *5*, 139–153.

Kirstetter, P., Anderson, K., Porse, B.T., Jacobsen, S.E., and Nerlov, C. (2006). Activation of the canonical Wnt pathway leads to loss of hematopoietic stem cell repopulation and multilineage differentiation block. Nat. Immunol. *7*, 1048–1056.

Krevvata, M., Silva, B.C., Manavalan, J.S., Galan-Diez, M., Kode, A., Matthews, B.G., Park, D., Zhang, C.A., Galili, N., Nickolas, T.L., et al. (2014). Inhibition of leukemia cell engraftment and disease progression in mice by osteoblasts. Blood *124*, 2834–2846.

Krivtsov, A.V., Twomey, D., Feng, Z., Stubbs, M.C., Wang, Y., Faber, J., Levine, J.E., Wang, J., Hahn, W.C., Gilliland, D.G., et al. (2006). Transformation from committed progenitor to leukaemia stem cell initiated by MLL-AF9. Nature *442*, 818–822.

Kusumbe, A.P., Ramasamy, S.K., and Adams, R.H. (2014). Coupling of angiogenesis and osteogenesis by a specific vessel subtype in bone. Nature *507*, 323–328.

Kusumbe, A.P., Ramasamy, S.K., Itkin, T., Mäe, M.A., Langen, U.H., Betsholtz, C., Lapidot, T., and Adams, R.H. (2016). Age-dependent modulation of vascular niches for haematopoietic stem cells. Nature *532*, 380–384.

Lassailly, F., Foster, K., Lopez-Onieva, L., Currie, E., and Bonnet, D. (2013). Multimodal imaging reveals structural and functional heterogeneity in different bone marrow compartments: functional implications on hematopoietic stem cells. Blood *122*, 1730–1740.

Law, C.W., Chen, Y., Shi, W., and Smyth, G.K. (2014). voom: precision weights unlock linear model analysis tools for RNA-seq read counts. Genome Biol. *15*, R29.

Liao, Y., Smyth, G.K., and Shi, W. (2014). featureCounts: an efficient general purpose program for assigning sequence reads to genomic features. Bioinformatics *30*, 923–930.

Liberzon, A., Birger, C., Thorvaldsdóttir, H., Ghandi, M., Mesirov, J.P., and Tamayo, P. (2015). The Molecular Signatures Database (MSigDB) hallmark gene set collection. Cell Syst. *1*, 417–425.

Lo Celso, C., Fleming, H.E., Wu, J.W., Zhao, C.X., Miake-Lye, S., Fujisaki, J., Côté, D., Rowe, D.W., Lin, C.P., and Scadden, D.T. (2009). Live-animal tracking of individual haematopoietic stem/progenitor cells in their niche. Nature *457*, 92–96.

Miraki-Moud, F., Anjos-Afonso, F., Hodby, K.A., Griessinger, E., Rosignoli, G., Lillington, D., Jia, L., Davies, J.K., Cavenagh, J., Smith, M., et al. (2013). Acute myeloid leukemia does not deplete normal hematopoietic stem cells but induces cytopenias by impeding their differentiation. Proc. Natl. Acad. Sci. USA *110*, 13576–13581.

Morrison, S.J., and Scadden, D.T. (2014). The bone marrow niche for haematopoietic stem cells. Nature *505*, 327–334.

Muzumdar, M.D., Tasic, B., Miyamichi, K., Li, L., and Luo, L. (2007). A global double-fluorescent Cre reporter mouse. Genesis *45*, 593–605.

Ossenkoppele, G.J., Stussi, G., Maertens, J., van Montfort, K., Biemond, B.J., Breems, D., Ferrant, A., Graux, C., de Greef, G.E., Halkes, C.J., et al. (2012). Addition of bevacizumab to chemotherapy in acute myeloid leukemia at older age: a randomized phase 2 trial of the Dutch-Belgian Cooperative Trial Group for Hemato-Oncology (HOVON) and the Swiss Group for Clinical Cancer Research (SAKK). Blood *120*, 4706–4711.

Padró, T., Ruiz, S., Bieker, R., Bürger, H., Steins, M., Kienast, J., Büchner, T., Berdel, W.E., and Mesters, R.M. (2000). Increased angiogenesis in the bone marrow of patients with acute myeloid leukemia. Blood *95*, 2637–2644.

Paic, F., Igwe, J.C., Nori, R., Kronenberg, M.S., Franceschetti, T., Harrington, P., Kuo, L., Shin, D.G., Rowe, D.W., Harris, S.E., and Kalajzic, I. (2009). Identification of differentially expressed genes between osteoblasts and osteocytes. Bone *45*, 682–692.

Passaro, D., Di Tullio, A., Abarrategi, A., Rouault-Pierre, K., Foster, K., Ariza-McNaughton, L., Montaner, B., Chakravarty, P., Bhaw, L., Diana, G., et al. (2017). Increased vascular permeability in the bone marrow microenvironment contributes to disease progression and drug response in acute myeloid leukemia. Cancer Cell *32*, 324–341.e6.

Pitt, L.A., Tikhonova, A.N., Hu, H., Trimarchi, T., King, B., Gong, Y., Sanchez-Martin, M., Tsirigos, A., Littman, D.R., Ferrando, A.A., et al. (2015). CXCL12-producing vascular endothelial niches control acute T cell leukemia maintenance. Cancer Cell *27*, 755–768.

Preibisch, S., Saalfeld, S., Schindelin, J., and Tomancak, P. (2010). Software for bead-based registration of selective plane illumination microscopy data. Nat. Methods *7*, 418–419.

Quail, D.F., and Joyce, J.A. (2013). Microenvironmental regulation of tumor progression and metastasis. Nat. Med. *19*, 1423–1437.

Ramasamy, S.K., Kusumbe, A.P., Wang, L., and Adams, R.H. (2014). Endothelial Notch activity promotes angiogenesis and osteogenesis in bone. Nature *507*, 376–380.

Ramasamy, S.K., Kusumbe, A.P., Schiller, M., Zeuschner, D., Bixel, M.G., Milia, C., Gamrekelashvili, J., Limbourg, A., Medvinsky, A., Santoro, M.M., et al. (2016). Blood flow controls bone vascular function and osteogenesis. Nat. Commun. *7*, 13601.

Rashidi, N.M., Scott, M.K., Scherf, N., Krinner, A., Kalchschmidt, J.S., Gounaris, K., Selkirk, M.E., Roeder, I., and Lo Celso, C. (2014). In vivo time-lapse imaging shows diverse niche engagement by quiescent and naturally activated hematopoietic stem cells. Blood *124*, 79–83.

Schepers, K., Pietras, E.M., Reynaud, D., Flach, J., Binnewies, M., Garg, T., Wagers, A.J., Hsiao, E.C., and Passegué, E. (2013). Myeloproliferative neoplasia remodels the endosteal bone marrow niche into a self-reinforcing leukemic niche. Cell Stem Cell *13*, 285–299.

Scott, M.K., Akinduro, O., and Lo Celso, C. (2014). In vivo 4-dimensional tracking of hematopoietic stem and progenitor cells in adult mouse calvarial bone marrow. J. Vis. Exp. e51683.

Somervaille, T.C., and Cleary, M.L. (2006). Identification and characterization of leukemia stem cells in murine MLL-AF9 acute myeloid leukemia. Cancer Cell *10*, 257–268.

Subramanian, A., Tamayo, P., Mootha, V.K., Mukherjee, S., Ebert, B.L., Gillette, M.A., Paulovich, A., Pomeroy, S.L., Golub, T.R., Lander, E.S., and Mesirov, J.P. (2005). Gene set enrichment analysis: a knowledge-based approach for interpreting genome-wide expression profiles. Proc. Natl. Acad. Sci. USA *102*, 15545–15550.

Tessier, P.A., Naccache, P.H., Clark-Lewis, I., Gladue, R.P., Neote, K.S., and McColl, S.R. (1997). Chemokine networks in vivo: involvement of C-X-C and C-C chemokines in neutrophil extravasation in vivo in response to TNF-alpha. J. Immunol. *159*, 3595–3602.

Waibel, M., Vervoort, S.J., Kong, I.Y., Heinzel, S., Ramsbottom, K.M., Martin, B.P., Hawkins, E.D., and Johnstone, R.W. (2017). Epigenetic targeting of Notch1-driven transcription using the HDACi panobinostat is a potential therapy against T-cell acute lymphoblastic leukemia. Leukemia. Published online September 15, 2017. https://doi.org/10.1038/leu.2017.282.

Wunderlich, M., Mizukawa, B., Chou, F.S., Sexton, C., Shrestha, M., Saunthararajah, Y., and Mulloy, J.C. (2013). AML cells are differentially sensitive to chemotherapy treatment in a human xenograft model. Blood *121*, e90–e97.

Xu, Y., Yuan, L., Mak, J., Pardanaud, L., Caunt, M., Kasman, I., Larrivée, B., Del Toro, R., Suchting, S., Medvinsky, A., et al. (2010). Neuropilin-2 mediates VEGF-C-induced lymphatic sprouting together with VEGFR3. J. Cell Biol. *188*, 115–130.

Zahiragic, L., Schliemann, C., Bieker, R., Thoennissen, N.H., Burow, K., Kramer, C., Zühlsdorf, M., Berdel, W.E., and Mesters, R.M. (2007). Bevacizumab reduces VEGF expression in patients with relapsed and refractory acute myeloid leukemia without clinical antileukemic activity. Leukemia *21*, 1310–1312.

STAR★METHODS

KEY RESOURCES TABLE

REAGENT or RESOURCE	SOURCE	IDENTIFIER
Antibodies		
Biotin anti-mouse CD3e	BioLegend	100304
Biotin anti-mouse CD4	BioLegend	100404
Biotin anti-mouse CD8a	BioLegend	100704
Biotin anti-mouse Ter119	BioLegend	116204
APC/Cy7 anti-mouse Ter-119	BioLegend	116223
APC/Cy7 anti-mouse CD45	BioLegend	103116
Biotin anti-mouse/human CD45R/B220	BioLegend	103204
Biotin anti-mouse Ly6G	BioLegend	127604
Biotin anti-mouse CD11b	BioLegend	101204
APC/Cy7 anti-mouse/human CD11b	BioLegend	101226
APC anti-mouse/human CD11b	BioLegend	101212
PE/Cy7 anti-mouse/human CD11b	BioLegend	101216
APC anti-mouse CD117 (c-Kit)	BioLegend	101212
APC/Cy7 anti-mouse CD117 (cKit)	BioLegend	105826
PerCP/Cy5.5 anti-mouse Ly6a/E (Sca-1)	BioLegend	108124
Anti-Mouse CD34 eFluor 660	eBioscience	50-0341-82
APC anti-mouse CD34	BioLegend	128612
PE-Cy7 anti-mouse CD16/32	BioLegend	101318
BV650 anti-mouse CD150 (SLAM)	BioLegend	115931
PE/Cy7 anti-mouse CD48	BioLegend	103424
Biotin anti-mouse CD31	BioLegend	102504
PE/Cy7 anti-mouse CD31	BioLegend	102524
Anti-Mouse Endomucin eFluor 660	eBioscience	50-5851-82
PE/Cy7 Streptavidin	eBioscience	405206
Streptavidin Pacific Orange	Invitrogen	S32365
Rat Anti-Mouse Endomucin antibody (V.7C7)	Santa-Cruz	sc-65495
Rabbit Anti-Laminin	Sigma-Aldrich	L9393
Rabbit anti-GFP	Abcam	ab6556
Goat anti-Rabbit IgG (H+L) Alexa Fluor 488	Thermo Scientific	A-11034
Goat anti-Rat IgG (H+L) Alexa Fluor 633	Thermo Scientific	A-21094
Anti-Human Factor VIII-Related Antigen	DAKO	N1505
Bacterial and Virus Strains		
pMSCV-MLL-AF9-IRESGFP	gift from Steve Lane (QIMR Barhofer, Brisbane) Somervaille & Cleary. Cancer Cell 10, 257-268 (2006).	N/A
NotchICNΔRamΔP	Gift from Jon C. Aster (Harvard Medical School) and Warren S. Pear (University of Pennsylvania) (Aster et al., 2000)	N/A
Biological Samples		
See table below in “Human trephine biopsies”	N/A	N/A
Chemicals, Peptides, and Recombinant Proteins		
Deferoxamine mesylate salt	Sigma-Aldrich	D9533
Tamoxifen	Sigma-Aldrich	T5648-1G

(*Continued on next page*)

Continued

REAGENT or RESOURCE	SOURCE	IDENTIFIER
Collagenase, Type I, powder	GIBCO	17018029
Cy5-Dextran 500 kD	Nanocs	DX500-S5-1
Recombinant Murine SCF	Peprotech	250-03
Recombinant Human IL-6	Peprotech	200-06
Recombinant murine TPO	Peprotech	315-14
Recombinant murine Flt-3 ligand	Peprotech	250-31L
StemSpan Serum Free Expansion Medium	STEMCELL Technologies	09650
ProLong Diamond Antifade Mountant	Thermo Scientific	P36961
BD Calibrite 3 Beads Unlabeled, FITC, PE, and PerCP Beads	BD Biosciences	340486
Vybrant DiD	Invitrogen	v22887
Streptavidin Microbeads	Miltenyi Biotech	130-048-101
Critical Commercial Assays		
Mouse VEGF DuoSet ELISA	R&D Systems	DY493
Mouse TNF-alpha DuoSet ELISA	R&D Systems	DY410
Mouse CXCL2/MIP-2 DuoSet ELISA	R&D Systems	DY452
NucBlue Fixed Cell ReadyProbes Reagent	Life Technologies	R37606
SYTO 17 Red Fluorescent Nucleic Acid Stain	Life Technologies	S7579
Deposited Data		
RNaseq data for endosteal AML cells, central AML cells and GMPs	GEO: GSE105159	https://www.ncbi.nlm.nih.gov/geo/query/acc.cgi?acc=GSE105159
Experimental Models: Cell Lines		
LinXE	Gift from H. Brady (Imperial College) (Dunne et al., 2010; Hannon et al., 1999).	N/A
Experimental Models: Organisms/Strains		
C57BL/6	Charles River	N/A
Flk1-GFP mice	gift from Alexander Medvinsky (University of Edinburgh) (Xu et al., 2010)	
PU1-YFP mice	gift from Claus Nerlov (University of Oxford) (Kirstetter et al., 2006)	
Col2.3-CFP mice	gift from David Rowe (University of Connecticut Health Center) (Paic et al., 2009)	
Col2.3-GFP mice	gift from David Rowe (University of Connecticut Health Center) (Hawkins et al., 2016)	
mT/mG mice	Muzumdar et al., 2007	
Fbxw7$^{lox/lox}$ Cdh5(PAC)-CreERT2$^{T/+}$ mice	Gift from R. Adams and A. Kusumbe	
Software and Algorithms		
Fiji/ImageJ	fiji.sc/	N/A
ZEN black	Zeiss	N/A
Definiens Developer 64	Definiens	N/A
Imaris	Bitplane	N/A
GraphPad Prism	GraphPad Software	N/A
FlowJo	Tree Star	N/A
Matlab	Mathworks	N/A

CellPress

CONTACT FOR REAGENT AND RESOURCE SHARING

Further information and requests for resources and reagents should be directed to and will be fulfilled by the Lead Contact, Cristina Lo Celso (c.lo-celso@imperial.ac.uk).

EXPERIMENTAL MODEL AND SUBJECT DETAILS

Mice

All animal work was in accordance with the animal ethics committee (AWERB) at Imperial College London, UK and UK Home Office regulations (ASPA, 1986). Flk1-GFP mice were a gift from Alexander Medvinsky (University of Edinburgh) (Xu et al., 2010), PU1-YFP were a gift from Claus Nerlov (University of Oxford) (Kirstetter et al., 2006). C57BL/6 mice were from Harlan UK Ltd; Col2.3-GFP (Hawkins et al., 2016), Col2.3-CFP (Paic et al., 2009), and mT/mG (Muzumdar et al., 2007) mice were bred and housed at Imperial College London, according to institutional guidelines. Animals were housed in a barrier facility, under a 14-hour light / 10-hour dark cycle and temperature-controlled environment with standard diet and water *ad libitum*. Male and female mice > 6 weeks old were used.

AML experimental model

To generate disease traceable by both flow cytometry and microscopy, GMPs were sorted from C57/B6 wild-type, mT/mG or PU.1-YFP mice. GMPs were transduced with pMSCV-MLL-AF9-GFP-based retroviruses as described in (Krivtsov et al., 2006) and then transplanted into sub-lethally irradiated mice. > 8 weeks post transplantation, recipient mice developed leukemia characterized by multi-organ infiltration of leukemic blasts. GFP^+ cells were then harvested from BM and spleen and stored. Blasts from each primary recipient were labeled as a separate batch. Primary blasts from different mice were thawed, suspended in phosphate balanced salt solution (PBS) and 100,000 viable cells were transplanted through tail vein injection into secondary, non-conditioned recipient mice. In some instances secondary blasts were used. Progressive blast expansion was observed from day 8-10 and full BM infiltration was typically reached between day 20 and 28, depending on the primary blasts analyzed. This was accompanied by infiltration of the spleen, typically delayed compared to BM infiltration.

T-ALL experimental model

T-ALL was generated as described in detail in (Hawkins et al., 2016; Hawkins et al., 2014). Briefly, fetal liver cells were transduced with NotchICNΔRamΔP-based retrovirus. 1×10^6 $DsRed^+$ fetal liver cells were transplanted into lethally irradiated recipients. Upon onset of primary disease (6-25 weeks), $DsRed^+$ blasts were harvested and cryopreserved. 10,000 primary blasts were transplanted into sub-lethally irradiated recipients, leading to full BM infiltration in 28 days. Secondary blasts were cryopreserved. 50,000 secondary blasts were thawed, Ficoll purified and injected into non-irradiated tertiary recipients used for experiments. Disease progression was monitored through sampling of peripheral blood. Mice at different stages of infiltration were sacrificed and bones harvested for immunofluorescence.

Human samples

Human trephine biopsies were obtained from patients after informed consent had been obtained, under full ethical approval by the Alfred Hospital, the Peter MacCallum Cancer Centre Human Research Ethics Committee, and St. Vincent's Hospital Melbourne. Information about control and AML samples is provided bellow.

	Control
#	Diagnosis
**1**	Primary CNS lymphoma
**2**	Diffusive large B cell lymphoma
**3**	Mantle cell lymphoma
**4**	Non-hodgkin lymphoma
**5**	Non-hodgkin lymphoma
**6**	Non-hodgkin lymphoma

	AML		
#	WHO classification	Cytogenetics	Blasts
**1**	Acute monoblastic and monocytic leukemia	t(10;11)	94%
**2**	Therapy-related acute myeloid leukemia	t(9;11)	88%
**3**	Acute promyelocytic leukemia	t(15;17)	83%

METHOD DETAILS

Drug treatments

The sample size required for animal experiments was based on preliminary data.

For DFO treatment, daily 100mg/kg deferoxamine mesylate (DFO; Sigma) was administered I.P. from day 8 until day 22 post-transplantation of AML blasts, at which time mice were sacrificed and their BM analyzed. Control mice were injected I.P. with 100μl of PBS.

Induction chemotherapy for AML was administered when BM infiltration was over 50% by injecting 100mg/kg cytarabine (Ara-C) I.V. for 5 days and 3mg/kg doxorubicin (Doxo) for 3 days. Ara-C was co-delivered with Doxo on days 1 to 3 and alone on days 4 and 5, mimicking the 7+3 regimen used in AML patients (Wunderlich et al., 2013). Both drugs were purchased from Sellekchem, MA or obtained from the Imperial College Healthcare NHS Trust.

For EC-specific deletion of Fbxw7, tamoxifen (500 μl/mouse I.P.; Sigma) was given daily to Fbxw7$^{lox/lox}$ Cdh5(PAC)-CreERT2$^{T/+}$ (Fbxw7$^{i\Delta EC}$) mice and to control Fbxw7$^{lox/lox}$ and WT mice. In experiments where relapse and survival were analyzed, tamoxifen was given daily between day 10 and 20 post-transplantation, at which point chemotherapy was initiated. Blinding was done for the tamoxifen studies.

BM chimeras

To generate chimeras, whole BM cells were obtained from femurs and tibia of wild-type or mT/mG donor mice, diluted in PBS and transplanted intravenously into lethally irradiated (two doses of 5.5 Gy separated by 3 hours) mT/mG, Col2.3-CFP or Flk1-GFP recipient mice at a dose of 1.5x10^6 cells/mouse. Mice were kept on baytril-treated water for five weeks following transplantation. > 95% chimerism was confirmed after 8 weeks and at that point mice were injected with AML cells and used for intravital imaging experiments.

Flow cytometry

For hematopoietic and leukemic cell analysis, bones were crushed in PBS with 2% fetal bovine serum and the cells, filtered through a 40 μm strainer and stained. In some instances, the metaphysis of tibias and femurs were separated and crushed, while the diaphysis was flushed. Cells from crushed metaphysis and flushed diaphysis were then stained and analyzed separately. For stroma analysis, tibias and femurs were crushed, digested with collagenase I (Worthington, UK) at 37°C, for 20min with 110rpm agitation, and the obtained cells were filtered through a 70 μm strainer and stained. The following fluorochrome-conjugated or biotinilated primary antibodies specific to mouse were used: CD3e (145-2C11), CD4 (GK1.5), CD8a (53-6.7), Ter119 (TER119), B220 (RA3-6B2), Ly6G (RB-68C5), CD11b (M1/70), cKit (2B8), Sca-1 (D7), CD34 (RAM34), CD16/32 (93), CD150 (TC15-12F12.2), CD48 (HM48-1), CD31 (MEC13.3) all from Biolegend, and endomucin (V.7C7) from eBiosciences. For secondary staining, streptavidin Pacific Orange (Invitrogen) and streptavidin PE Cy7 (Biolegend) were used. Live and dead cells were distinguished using 4,6-diamidino-2-phenylindole (DAPI, Invitrogen). To detect nucleic acids, the cell-permeant SYTO® 17 Red Fluorescent Nucleic Acid Stain (Molecular Probes) was used. Calibrite Beads (BD Biosciences) were used to determine absolute cell counts, as previously described (Hawkins et al., 2007). Cells were analyzed with a LSR Fortessa, sort-purified using a FACSAria III (BD Biosciences) and data were analyzed with FlowJo (Tree Star).

HSC transplantation and homing analysis

Tibias, femurs, ileac bones, vertebrae and sternum were harvested from donor mice, crushed and filtered through a 40 μm strainer. Whole BM cells were labeled with a cocktail of biotinilated lineage antibodies (CD3, CD4, CD8, Ter119, B220, Ly6G, CD11b) and streptavidin magnetic Microbeads (Miltenyi Biotech) to perform a lineage depletion using the MACS® Column Technology (Miltenyi Biotech). The lineage-depleted sample was stained and sorted for phenotypic HSCs, defined as lineage$^-$cKit$^+$Sca-1$^+$CD48$^-$CD150$^+$ cells. HSCs were centrifuged, suspended in PBS and incubated with Vybrant® 1,1'-dioctadecyl-3,3,3′-tetramethylindodicarbocyanine perchlorate (DiD) (ThermoFisher Scientific) for 10 min, at 37°C. After washing, 5,000 to 12,000 DiD-labeled HSCs were transplanted per mouse via tail vein injection. Control and leukemic recipient mice had been previously lethally irradiated (two irradiations of 5.5Gy, 3 hours apart). 2 days after transplantation, recipient mice were sacrificed and femurs, tibias and the spleen harvested. DiD$^+$DAPI$^-$cKit$^+$Sca-1$^+$CD48$^-$CD150$^+$ cells were detected by flow cytometry.

LKS culture and DFO *in vitro* treatment

Lineage depletion was performed as described above. Live lineage$^-$cKit$^+$Sca-1$^+$ (LKS) cells were sorted and plated at a density of 15.000 cells per well in a 48-well plate. Cells were maintained in StemSpan media (Stem Cell Technologies) with added 50ng/μl SCF, 10ng/μl IL-6, 10ng/μl TPO and 20ng/μl Flt3 (all from Peprotech). LKS cells were incubated with 0.5μM, 5μM, 50μM and 500μM DFO. After two days, cells were harvested, stained and the number and frequency of DAPI$^-$cKit$^+$Sca-1$^+$CD48$^-$CD150$^+$ HSCs analyzed by flow cytometry.

RNA sequencing and analysis

RNA-seq was performed as described previously (Waibel et al., 2017, In Press, *Leukaemia*). Mice were transplanted with primary AML cells from 3 different donors (batch_1_BM_mTmG, batch_3_BM_mTmG and batch_19_spleen_mTmG). Upon full infiltration,

tibias and femurs were harvested and metaphysis and diaphysis separated using scissors. Endosteal AML cells were isolated by crushing the metaphysis and sorting $DAPI^{-}GFP^{+}mTomato^{+}$ cells. Central AML cells were isolated by flushing the diaphysis and sorting $DAPI^{-}GFP^{+}mTomato^{+}$ cells. The control population, GMPs, was sorted from whole BM of age- and sex-matched wild-type mice. Total RNA was extracted using RNeasy® Mini Kit (QIAGEN, Hilden, Germany). The extracted RNA was analyzed on the Agilent 4200 Tapestation prior to library preparation. High quality RNA with RIN values greater than 9 was used for downstream application. 3′mRNA-sequencing libraries were prepared from 100ng of total RNA using the QuantSeq 3′ mRNA-Seq Library Prep Kit (Lexogen) according to the manufacturers instructions and sequenced on a NextSeq 500 (Illumina). The single-end 75bp were demultiplexed using CASAVAv1.8.2 and Cutadapt (v1.9) was used for read trimming. The trimmed reads were subsequently mapped to the mouse genome (mm10) using HISAT2. FeatureCounts was used for read counting (Liao et al., 2014) after which differential gene expression analysis was performed using Voom-LIMMA packages (Law et al., 2014). GSEA2-2.2.2 was used for Gene set enrichment analysis (GSEA) (Liberzon et al., 2015; Subramanian et al., 2005).

Enzyme-linked immunosorbent assay (ELISA)

To obtain BM supernatants, tibias and femurs were harvested from control and AML-burdened mice. With scissors, the metaphysis and diaphysis of long bones were separated. To obtain *flushed BM supernatant,* 70μl of PBS were flushed through each diaphysis, collected and reflushed; then, cells were excluded by centrifugation at 400 g for 5min; the supernatant was collected and any remaining cells excluded by centrifugation at 500 g for 5min. To obtain *crushed BM supernatant*, the metaphyses were gently crushed in 150μl of PBS, and the supernatant isolated by centrifugation as described above. Serum was prepared by collecting blood through cardiac puncture after terminal anesthesia with pentobarbital; blood was then left at 4°C for 3 hours or more to allow clot formation and centrifuged at 12,000 g for 10min at 4°C; the supernatant (serum) was then transferred to a new eppendorf tube. BM supernatants and serum were stored at −20°C until used for ELISA. DuoSet ELISAs (R&D Systems) were performed according to the manufacturer's instructions.

Intravital microscopy

Intravital microscopy was performed using a Zeiss LSM 780 upright confocal microscope equipped with Argon (458, 488 and 514 nm), a diode-pumped solid-state 561 nm laser and a Helium-Neon 633 nm, a tunable infrared multiphoton laser (Spectraphysics Mai Tai DeepSee 690-1020 nm), 4 non-descanned detectors (NDD) and an internal spectral detector array. In some cases a Leica SP5 was used instead. Live imaging of the calvarium BM was done as described in (Hawkins et al., 2016) and (Scott et al., 2014). The spleens of live mice were imaged under general, terminal anesthesia. Anesthesia was induced and maintained with isoflurane in medical O_2 (4% isoflurane in 4L/min O_2 for induction and 1%–2% isoflurane in 1L/min O_2 for maintenance), throughout the procedure. Mice were placed in the right lateral decubitus position and a small section of hair was removed from the left flank. A 5-8mm abdominal incision on the left flank above the spleen was used to expose the surface of the spleen, which was mechanically stabilized with a gentle vacuum using a coverslip vacuum chamber similar to that used in (Headley et al., 2016). A drop of water was placed on top of the chamber coverslip and the spleen was imaged using a long working distance W Plan-Apochromat × 20 DIC water immersion lens (1.0 N.A.). Blood vessels were labeled with 80 μL of 8mg/ml 500kD Cy5-Dextran (Nanocs, MA).

Second harmonic signal was excited at 860-880nm and detected with external detectors. CFP signal was excited at 870nm or 458nm and detected using external or internal detectors; GFP signal: excitation at 880nm or 488nm, external or internal detectors; YFP signal: excitation at 488nm or 514nm, internal detectors. mTomato/DsRed and Cy5 signals were respectively excited at 561nm and 633nm and detected using internal detectors. To simultaneously detect SHG, CFP, mTomato, YFP and Cy5 signals in chimeras, lambda acquisition and online fingerprinting were used and the signal collected using an external 32-channel gallium arsenide phosphide (GaAsP) detector. The reference spectra were acquired in different BM areas of the same mouse.

Immunofluorescence of undecalcified long bone sections

Tibias, femurs and hips were harvested and fixed overnight in periodate-lysine-paraformaldehyde (PLP) fixative, at 4°C. The bones were then washed with 0.1M phosphate buffer, immersed in sucrose (10%–30% gradient) for 48h for cryoprotection, frozen in optimal cutting temperature (OCT) compound (TissueTek) and kept at −80°C. Sections were obtained using a cryostat (Leica) and the Cryojane tape transfer system (Leica microsystems). Slides were either kept at 4°C and used in the following week or stored at −80°C. For staining, slides were re-hydrated in PBS, permeabilised in 0.1% Triton X-100, blocked in 5% goat serum and incubated with primary antibodies overnight, at 4°C. After washing in PBS, slides were incubated with secondary antibodies, counter-stained with DAPI, washed in 0.1% Triton X-100 and mounted with Prolong Diamond antifade (Invitrogen). The following antibodies were used: endomucin (V.7C7, Santa Cruz, 1:100), laminin (L9393, Sigma-Aldrich, 1:50), GFP (ab6556, Abcam, 1:500), goat anti-rat IgG Alexa Fluor 488 (Life Technologies, 1:400), goat anti-rabbit IgG Alexa Fluor 633 (Life Technologies, 1:400). Images were obtained using a Zeiss LSM 780 upright confocal/two-photon combined microscope (see intravital microscopy section) and analyzed using Fiji/ImageJ.

Human trephine biopsies

De-waxed human trephine biopsy sections, obtained under ethical approval from Alfred Hospital, Melbourne, were stained with vWF antibody (Ready-to-Use, DAKO), counterstained and mounted for viewing. Representative areas of each section were captured and analyzed by two independent researchers.

Image processing and quantification

ZEN black (Zeiss, Germany) software was used to stich three-dimensional BM and spleen tilescans (tilescans represent individual tiles stitched together to form a composite). FIJI/ImageJ was used to visualize, register (Preibisch et al., 2010) and process raw data. FIJI was used to manually crop out autofluorescent signal from out of the tissue. Cell tracking was performed using FIJI and cells enumerated in the ROI manager. Automated cell segmentation, and volume measurements were performed in Definiens (Definiens Developer 64, Germany) using local heterogeneity segmentation (Khorshed et al., 2015) to isolate Flk1-GFP$^+$, Col2.3-GFP$^+$ and mTomato$^+$ AML cells. Vessel-bone colocalization was analyzed using Imaris (Bitplane, Switzerland). After creating a surface for Flk1-GFP$^+$ signal and a surface for bone (SHG) signal from half tilescans, the Imaris XTension "Surface surface colocalization" was used.

Endosteal vessels were quantified in immunofluorescence sections by dividing the length of blood vessels (marked with laminin and endomucin) in contact with the bone surface (SHG signal) by the total length of the endosteal surface. Metaphyseal and diaphysial vessels were quantified by thresholding the vascular signal in the metaphysis and diaphysis and quantifying the area occupied by blood vessels. Microvascular density was quantified manually counting blood vessels and dividing the obtained counts by the total area.

Vessels oscillation was quantified in FIJI. After registration, maximum projections of 3D movies were produced. In each movie, 2 regions of interest (ROI) were selected and combined in a single object. After clearing the outside of ROIs, a Gaussian filter and a bleach correction were applied and the vessel movement automatically tracked using the plugin TrackMate.

QUANTITATION AND STATISTICAL ANALYSES

Raw data was visualized and processed using Microsoft Excel, MATLAB and GraphPad Prism (GraphPad Software Inc.). Group means were compared using the unpaired Student's t test. For multiple comparisons, one-way ANOVA with post hoc Tukey test or Bonferroni correction was used.

An exact one-tailed permutation test was implemented in MATLAB for the time-course data in Figure 7F. The statistic used was the sum across days of the difference between the mean infiltration in the Cre$^-$ and Cre$^+$ cohorts.

For all data, differences were considered significant whenever $p < 0.05$. * $p < 0.05$; ** $p < 0.01$; *** $p < 0.001$; **** $p < 0.0001$. Specific statistical parameters (e.g., number of animals used) can be found in the figure legends.

DATA AND SOFTWARE AVAILABILITY

The accession number for the RNA-seq data reported in this paper is GEO: GSE105159.

Cell Stem Cell, Volume *22*

Supplemental Information

Inhibition of Endosteal Vascular Niche Remodeling Rescues Hematopoietic Stem Cell Loss in AML

Delfim Duarte, Edwin D. Hawkins, Olufolake Akinduro, Heather Ang, Katia De Filippo, Isabella Y. Kong, Myriam Haltalli, Nicola Ruivo, Lenny Straszkowski, Stephin J. Vervoort, Catriona McLean, Tom S. Weber, Reema Khorshed, Chiara Pirillo, Andrew Wei, Saravana K. Ramasamy, Anjali P. Kusumbe, Ken Duffy, Ralf H. Adams, Louise E. Purton, Leo M. Carlin, and Cristina Lo Celso

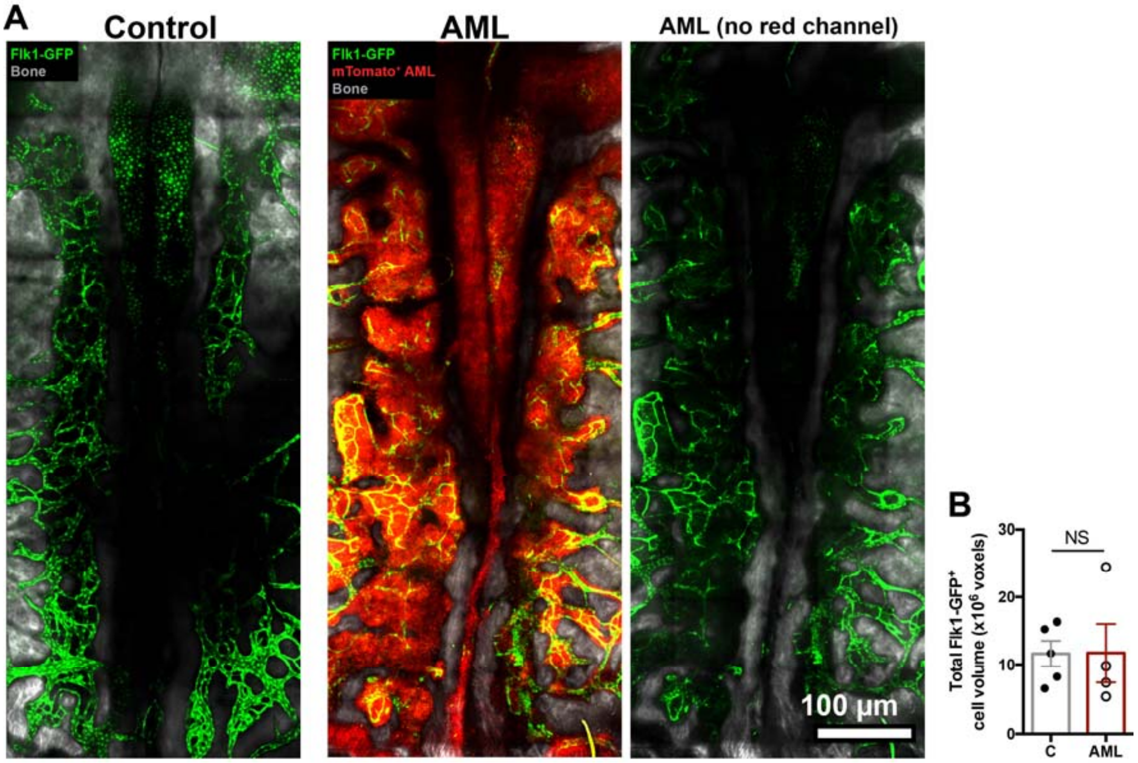

Figure S1 – Intravital imaging of Flk1-GFP mice, related to Figure 2. (A) Representative tilescans (composite of individual tiles) of control and leukemic Flk1-GFP mice. Green: Flk1-GFP^{+} cells (maximum projection); Grey: bone collagen SHG (median projection); Red: mTomato^{+} leukemia cells. **(B)** Total Flk1-GFP^{+} cell volume from acquired tilescans. Data are from 5 control and 4 AML mice.

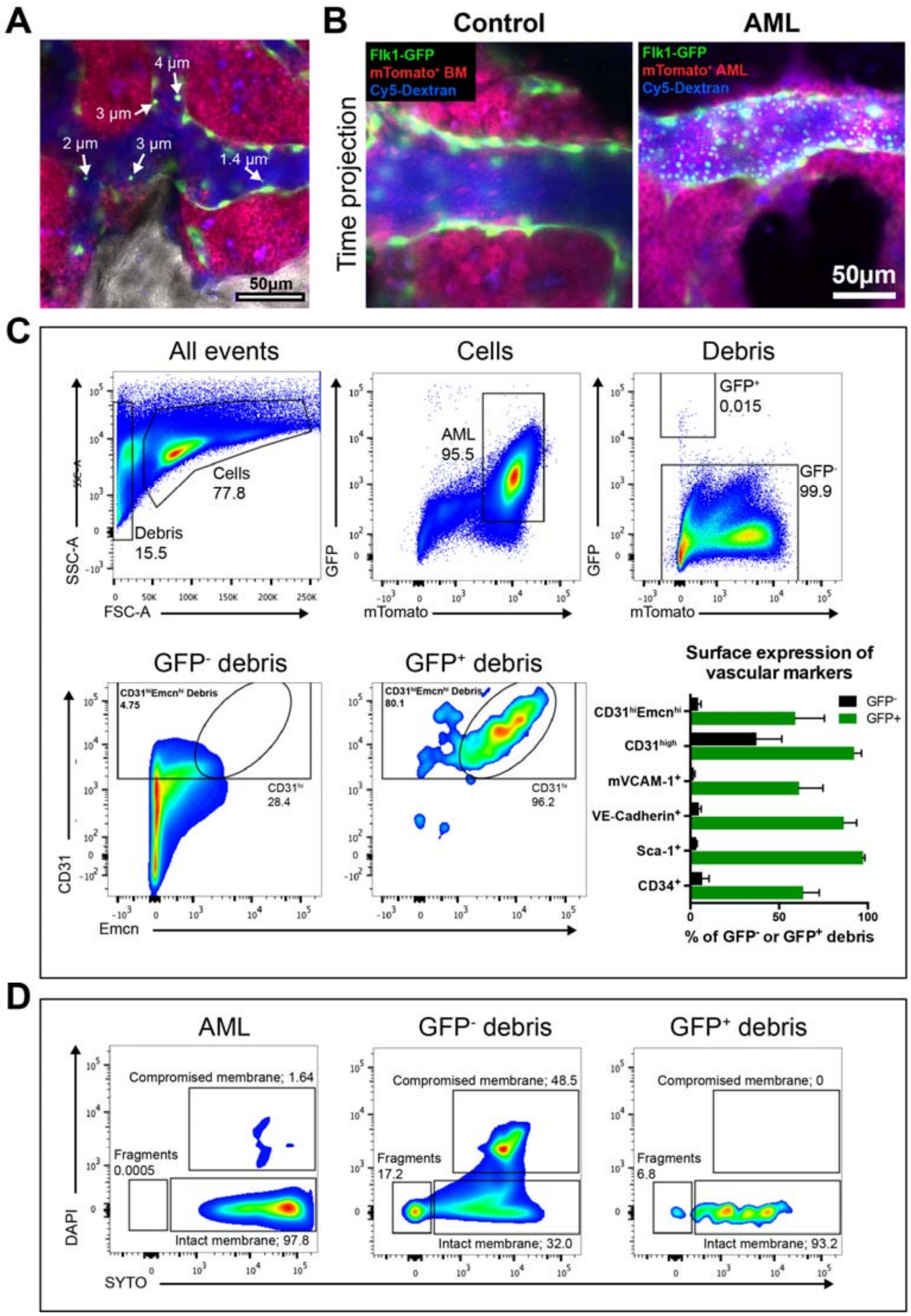

Figure S2 – Cellular debris found in circulation in Flk1-GFP$^+$ AML-burdened mice, related to Figure 2. (A, B) IVM images of GFP$^+$ debris found in circulation in leukemia-burdened, but not healthy Flk1-GFP mice (A: single 2D frame; B: time projection of Movie S3). **(C)** Gating strategy used to identify GFP$^+$ debris, expressing high levels of vascular markers, as assessed by flow cytometry of the BM. **(D)** GFP$^+$ debris is positive for the cell-permeable SYTO and negative for the cell-impermeable DAPI dyes, suggesting that they are membrane-layered particles, carrying variable levels of nucleic acids. Data representative of 3 leukemic mice.

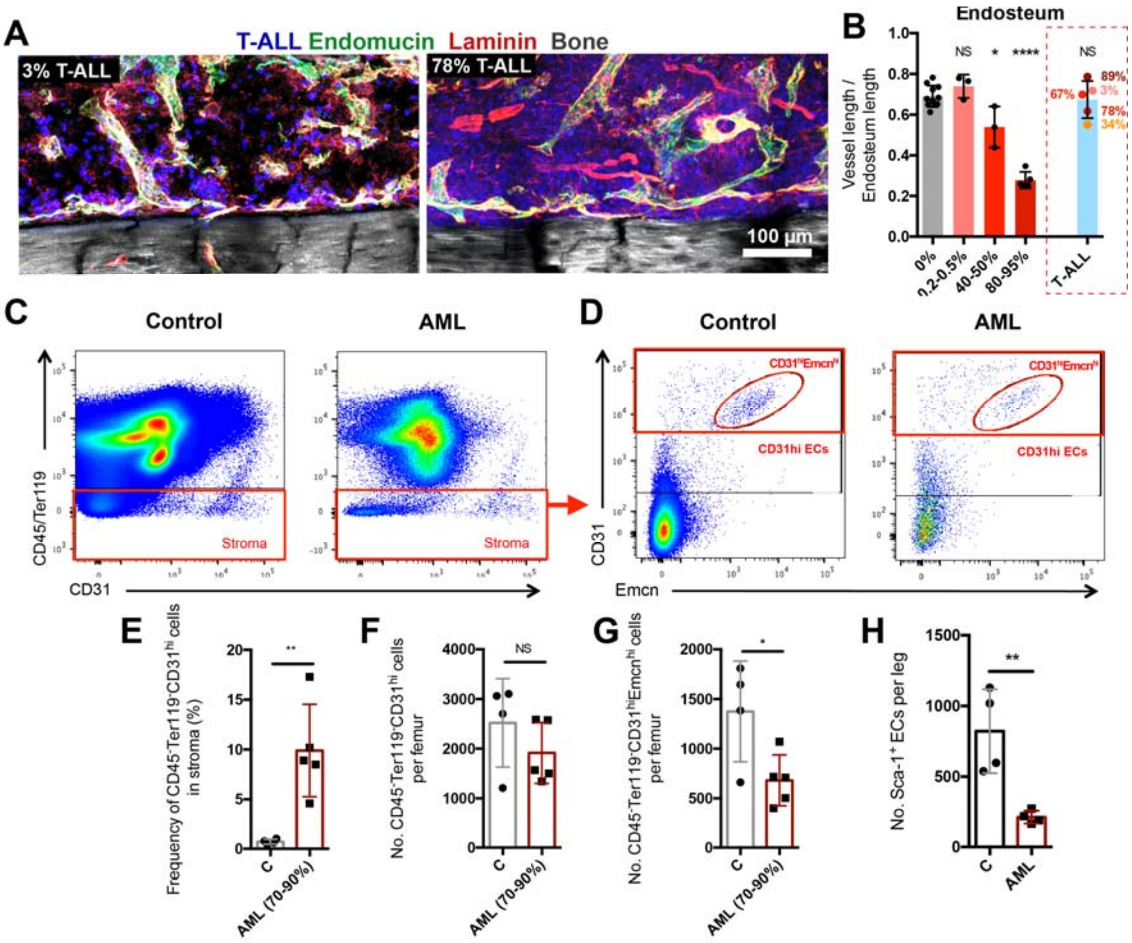

Figure S3 – Endosteal vessels are maintained in T-ALL but decreased in AML-burdened mice, related to Figure 3. (A) Maximum intensity projections of representative immunostained sections comparing endosteal vessels in mice poorly (3%) and highly (78%) infiltrated with T-ALL. Grey: bone collagen SHG; green: endomucin^{+} vessels; red: laminin^{+} vessels and extracellular matrix; blue: T-ALL cells. **(B)** Quantification of endosteal vessels in mice with different levels of T-ALL (each dot represents a mouse). Control and AML samples are reproduced from Figure 3C. **(C, D)** Gating strategy used to quantify BM stroma cells **(C)** and ECs **(D)** by flow cytometry. Although the frequency **(E)** of ECs is increased, absolute EC numbers **(F)** are unaltered in AML-burdened mice. Absolute numbers of **(G)** CD31^{hi}Emcn^{hi} and **(H)** CD31^{+}Sca-1^{+} endosteal ECs are significantly decreased in fully infiltrated mice. Data obtained from 4 control and 5 leukemic mice (C-G) and from 4 control and 4 leukemic mice (H). Error bars: mean ± SEM.

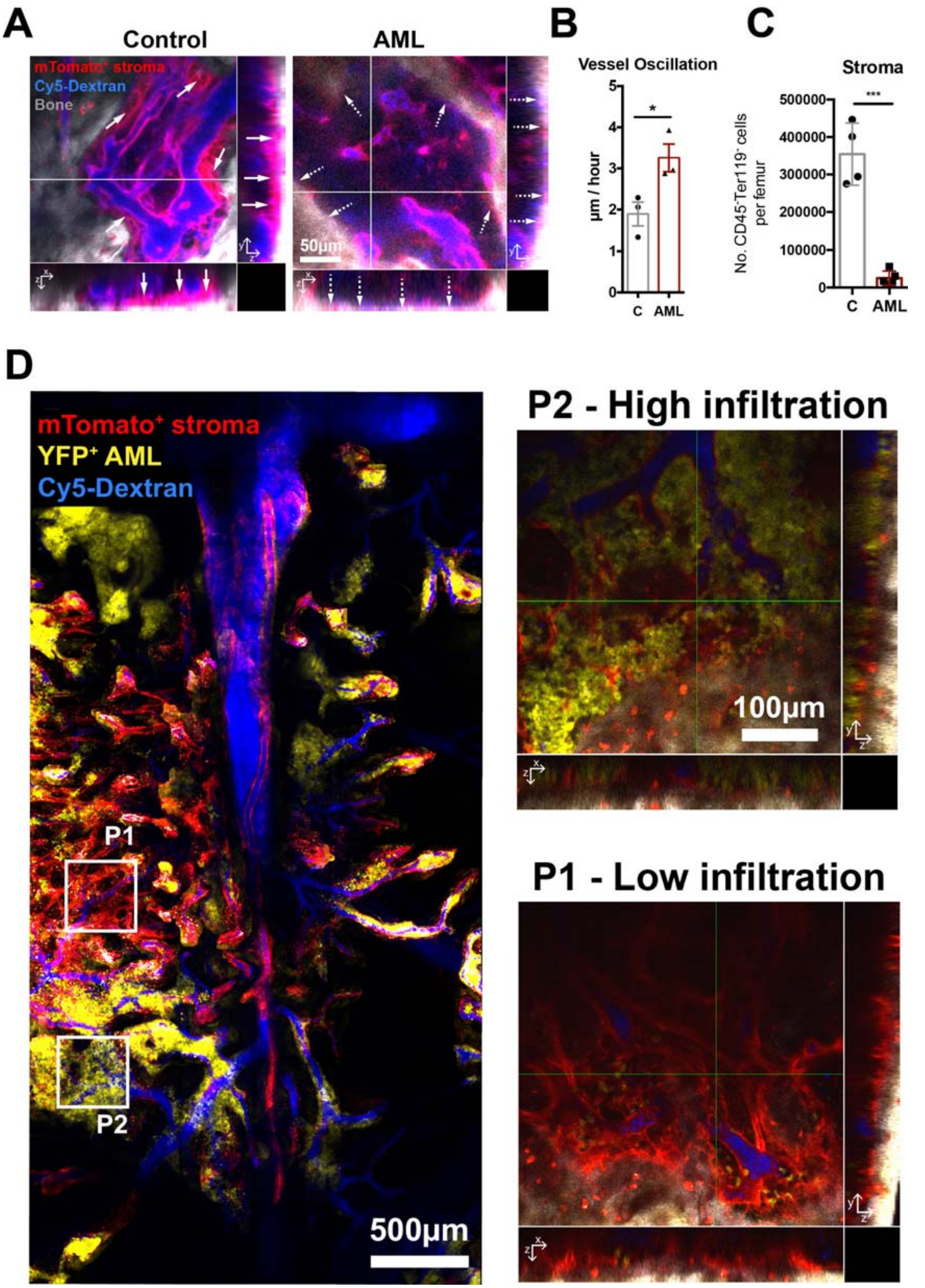

Figure S4 – Stroma is locally and progressively remodeled in AML, related to Figure 3. (A) Maximum intensity projections and orthogonal views of representative BM areas of mT/mG mice reconstituted with non-fluorescent wild-type BM and subsequently transplanted with YFP$^+$GFP$^+$ leukemia cells (AML, cells not shown).

AML-burdened mice show depletion of $mTomato^+$ stromal cells (red) adjacent to vessels (Cy-5 dextran; blue) and bone (SHG; grey). Arrows point at endosteal stroma in control mice. Dashed arrows evidence the loss of endosteal stroma in AML. Data representative of 3 control and 3 leukemic mice. YFP^+ AML cells not shown for clarity purposes. **(B)** Vascular oscillation is significantly increased in mice with AML, as shown in the Movie S4. Data pooled from a total of 2 BM positions per mouse, obtained from 3 control and 3 leukemic mice. Error bars: mean ± SEM **(C)** Absolute numbers of overall $CD45^-Ter119^-$ stromal cells are significantly decreased in AML-burdened mice. Data obtained from non-chimeric 4 control and 5 leukemic mice. *** $p<0.001$. Error bars: mean ± SD. **(D)** Representative tilescan (composite of individual tiles) and selected P1 and P2 areas showing AML cells (yellow) colonizing the calvarium in clusters and locally depleting mTomato+ stromal cells (red) adjacent to blood vessels (Cy5 dextran; blue) and bone (SHG; grey). Data representative of 3 mice.

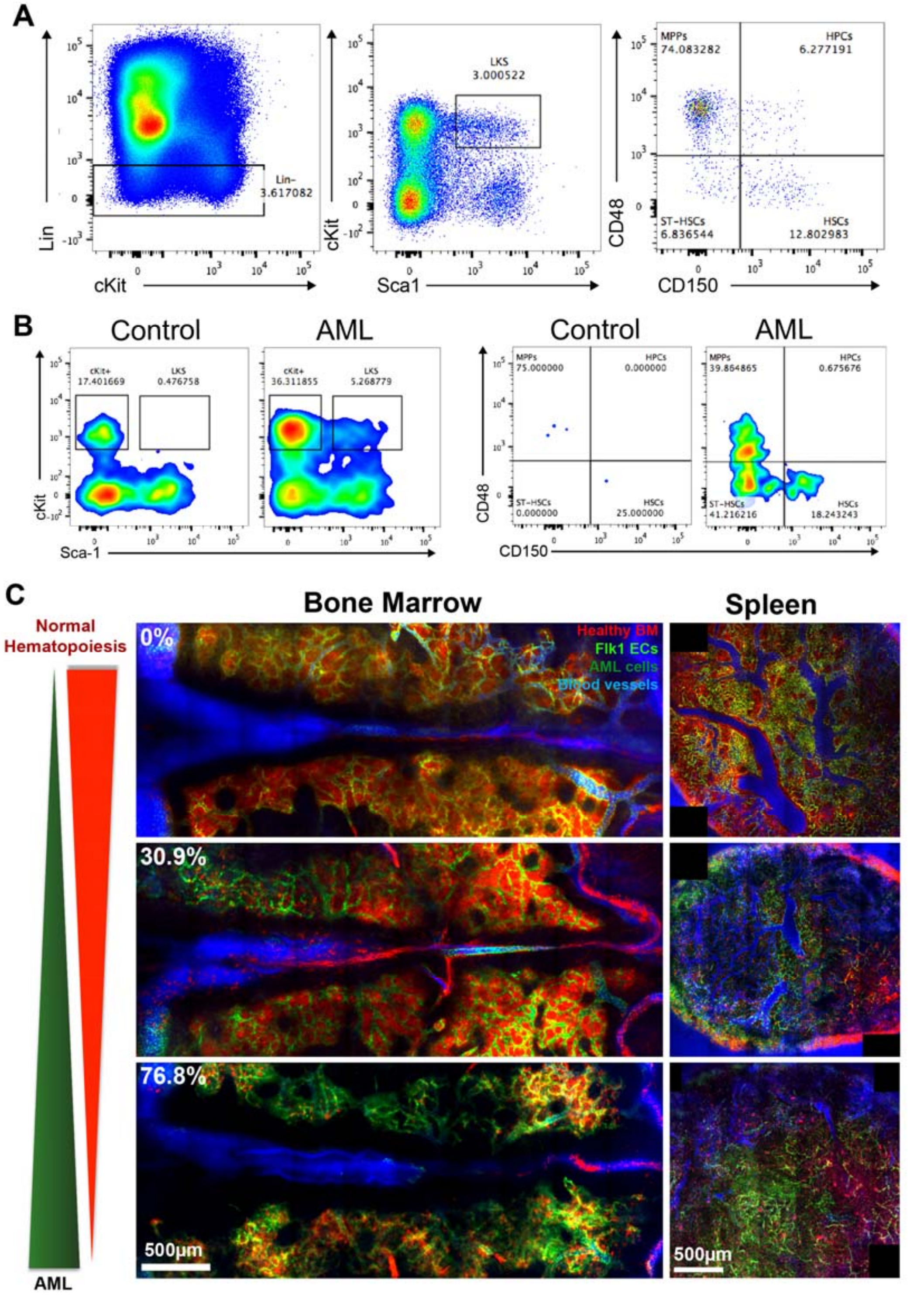

Figure S5 – Hematopoiesis loss in the BM is coupled with extramedullary hematopoiesis in the spleen, related to Figure 5. (A) Gating strategy used to identify healthy Lineage⁻ cKit⁺ Sca-1⁺ (LKS) progenitors and LKS CD48⁻ CD150⁺ hematopoietic stem cell (HSCs). **(B)** Flow cytometry analysis reveals emergence of LKS and HSC populations in the leukemic spleen. **(C)** Intravital imaging of BM and spleen. Maximum intensity projections of representative BM and spleen tilescans

(composite of individual tiles) of Flk1-GFP mouse chimeras reconstituted with mTomato^{+} hematopoietic cells and transplanted with GFP^{low} AML cells. In the case of spleen tilescans, some peripheral tiles are not included in the composite. mTomato^{+} hematopoietic cells are lost with increasing levels of AML infiltration. For each mouse, BM and spleen were imaged on the same day. To track single mTomato^{+} healthy hematopoietic cells in both the BM and spleen, we performed time-lapse imaging with high temporal resolution (30s acquisition interval) – see Figure 5. Green: Flk1-GFP^{+} ECs; red: mTomato^{+} healthy hematopoietic cells; blue: Cy5 Dextran.

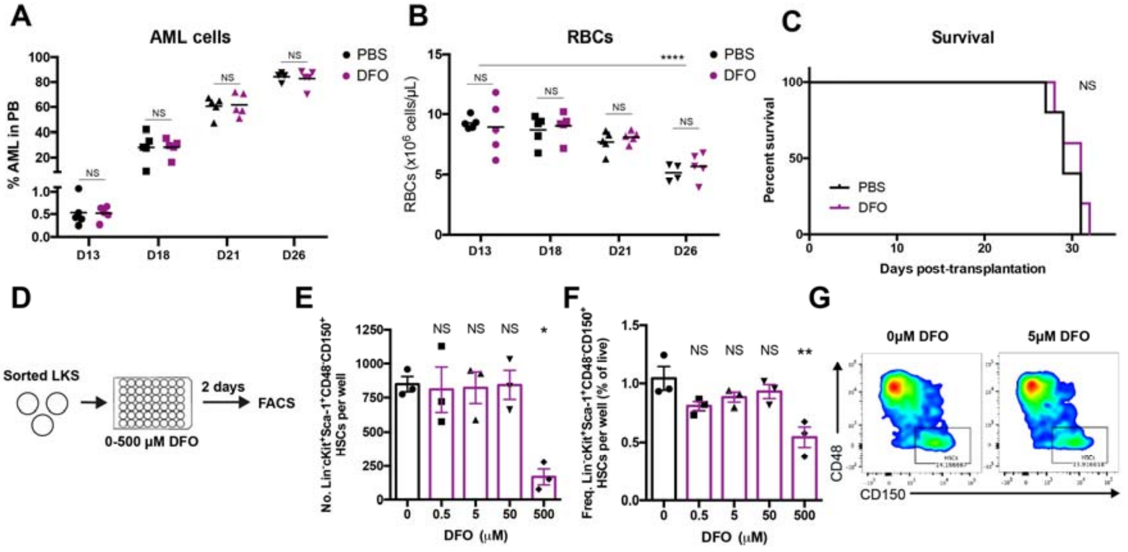

Figure S6 – DFO effect on AML progression *in vivo* and HSCs *in vitro*, related to Figure 6. Mice transplanted with AML were treated with either PBS or DFO from day 8 post-transplantation. **(A)** Percentage of mTomato^{+} blasts and **(B)** red blood cell (RBC) counts in the peripheral blood and **(C)** survival of DFO- and PBS-treated mice show similar disease progression in the two groups. Data obtained from 5 control (PBS) and 5 DFO-treated mice. **(D)** Live Lin^{-}cKit^{+}Sca-1^{+} (LKS) cells were sorted, plated and incubated with increasing concentrations of DFO. 2 days later the **(E)** number and **(F, G)** frequency of Lin^{-}cKit^{+}Sca-1^{+}CD48^{-}CD150^{+} HSCs were assessed by FACS. Data shown are representative of three independent experiments.

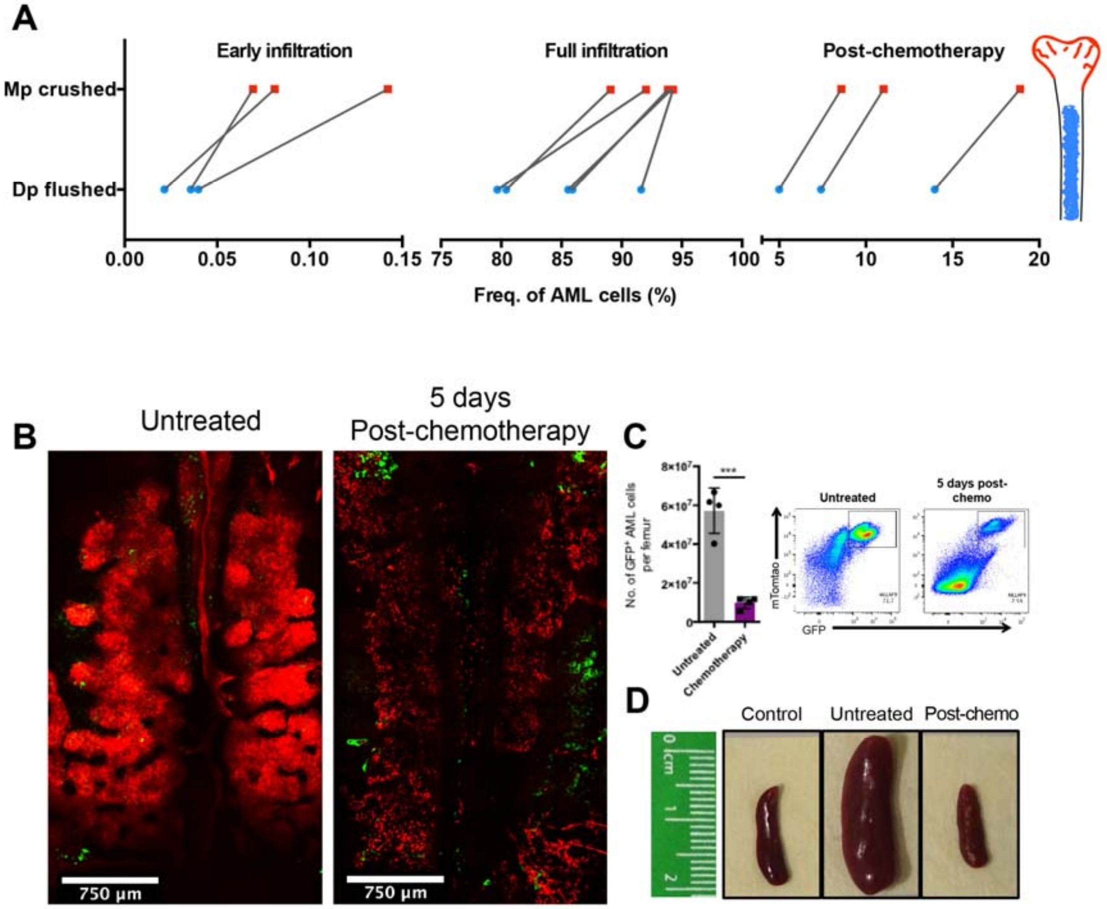

Figure S7 – Experimental model of AML induction chemotherapy, related to Figure 7. (A) Paired comparison shows that leukemic cells are more frequent in crushed metaphysis (Mp) than in flushed diaphysis (Dp) at very early (3 mice) and late (5 mice) infiltration and post-chemotherapy (3 mice). **(B)** Maximum projection tilescan (composite of individual tiles) of calvarium BM of Col2.3-GFP mice (green: osteoblastic cells) carrying mTomato^{+} AML cells (red) before and after chemotherapy (3 days of cytarabine and doxorubicin followed by 2 days of cytarabine alone). **(C)** Flow cytometry quantification of absolute numbers of AML cells in the BM. Data obtained from 4 control and 4 leukemic mice. Error bars: mean ± S.D. **(D)** Appearance of spleens from control, untreated and 5 days-treated mice.

Supplementary movies legends

Movie S1 – Inefficient angiogenesis in AML-burdened mice. Representative maximum projection of 3D time-lapse data (shown at 10 frames per second) of an area from a control (left) and leukemic (right) Flk1-GFP mouse collected every 90s for 90min (shown in figure 2G). Red arrowheads point to vascular sprouts that rapidly retract. Black: Flk1-GFP^{+} cells. Representative of 4 control and 3 leukemic mice.

Movie S2 – Blood vessel fragmentation in mice with AML. Maximum projection of 3D time-lapse data (shown at 10 frames per second) of vascular disintegration (circled area; red arrows) in a leukemic Flk1-GFP mouse. Filmed every 90s for 90min. Black: Flk1-GFP^{+} cells. Equivalent events were never observed in control mice.

Movie S3 – Circulating endothelial debris in BM vessels of AML-burdened mice. Representative 2D time-lapse data collected every 156ms for 4min 24s (shown at 5 frames per second) from a Flk1-GFP mouse reconstituted with mTomato^{+} healthy hematopoietic cells (left; Control) and a Flk1-GFP mouse infiltrated with mTomato^{+} AML (right; AML). In control mice (left) no debris particles are detected in circulation but in leukemic mice (right) frequent endothelial debris is found inside the vascular lumen, sometimes adhering to the endothelium. Green: GFP signal; red: mTomato^{+} healthy hematopoietic cells (left) or AML cells (right); blue: Cy5-Dextran. Arrowheads follow some of the debris observed in circulation. Representative of 3 control and 4 leukemic mice.

Movie S4 - Stroma dynamics in mice with AML. Representative maximum projections of 3D time-lapse data (shown at 10 frames per second) collected at ten-minute intervals for 7h and 20min from a mT/mG control (left) chimera and a mT/mG chimera with high infiltration of GFP^{+}YFP^{+} AML (right). AML cells not shown for clarity purposes. Red: mTomato^{+} stromal cells; blue: Cy-5 dextran^{+} blood vessels. Arrows follow oscillating vessels in AML-burdened mouse. Representative of 3 control and 3 leukemic mice.

Movie S5 – Cell adhesion to the splenic endothelium. Representative maximum projections of time-lapse data (shown at 7 frames per second) of 2 areas scanned every 30s for 15min from the spleen of a leukemic Flk1-GFP mouse with mTomato^{+} residual healthy hematopoietic cells. In area 1, the arrow points to a healthy hematopoietic cell adhering statically to the endothelium. In position 2, a cell adheres, crawls and detaches from the endothelium. Green: Flk1^{+} GFP ECs; red: mTomato^{+} healthy hematopoietic cells; blue: Cy5-Dextran.

Movie S6 – Transendothelial migration in the bone marrow. Representative maximum projections of time-lapse data (shown at 7 frames per second) of 2 areas scanned every 30s for 15min from the BM of a leukemic Flk1-GFP mouse with mTomato^{+} residual healthy hematopoietic cells. In position 1, the arrow points to a normal hematopoietic cell intravasating and leaving the BM. In position 2, a cell adheres and extravasates towards the tissue. Green: Flk1^{+} GFP ECs; red: mTomato^{+} non-malignant hematopoietic cells; blue: Cy5-Dextran.

Movie S7 – Turbulent blood flow in vessels within AML infiltrated BM. Representative maximum projection (shown at 7 frames per second) of a vascular

bifurcation area collected every 30s for 15min from the spleen of a healthy (left) and a leukemic (right) Flk1-GFP mouse. Dark circles: AML cells form intravascular clusters that adhere to the endothelium and block blood flow; Green: $Flk1^+$ GFP ECs; Red: $mTomato^+$ non-malignant hematopoietic cells; Yellow: Cy5-Dextran.
